# Isoprenoid Alcohols are Susceptible to Oxidation with Singlet Oxygen and Hydroxyl Radicals

**DOI:** 10.1007/s11745-015-4104-y

**Published:** 2015-12-30

**Authors:** Joanna Komaszylo née Siedlecka, Magdalena Kania, Marek Masnyk, Piotr Cmoch, Iwona Lozinska, Zbigniew Czarnocki, Karolina Skorupinska-Tudek, Witold Danikiewicz, Ewa Swiezewska

**Affiliations:** Department of Lipid Biochemistry, Institute of Biochemistry and Biophysics, Polish Academy of Sciences (PAS), Pawinskiego 5a, 02-106 Warsaw, Poland; Laboratory of Mass Spectrometry, Institute of Organic Chemistry, Polish Academy of Sciences (PAS), Kasprzaka 44/52, 01-224 Warsaw, Poland; Faculty of Chemistry, University of Warsaw, Warsaw, Poland

**Keywords:** Isoprenoid oxidation, Singlet oxygen, Hydrogen peroxide, Sodium molybdate, Porphyrin, Hydroxyl radical, Oxygen, UV irradiation

## Abstract

Isoprenoids, as common constituents of all living cells, are exposed to oxidative agents—reactive oxygen species, for example, singlet oxygen or hydroxyl radicals. Despite this fact, products of oxidation of polyisoprenoids have never been characterized. In this study, chemical oxidation of isoprenoid alcohols (Prenol-2 and -10) was performed using singlet oxygen (generated in the presence of hydrogen peroxide/molybdate or upon photochemical reaction in the presence of porphyrin), oxygen (formed upon hydrogen peroxide dismutation) or hydroxyl radical (generated by the hydrogen peroxide/sonication, UV/titanium dioxide or UV/hydrogen peroxide) systems. The structure of the obtained products, hydroxy-, peroxy- and heterocyclic derivatives, was studied with the aid of mass spectrometry (MS) and nuclear magnetic resonance (NMR) methods. Furthermore, mass spectrometry with electrospray ionization appeared to be a useful analytical tool to detect the products of oxidation of isoprenoids (ESI–MS analysis), as well as to establish their structure on the basis of the fragmentation spectra of selected ions (ESI–MS/MS analysis). Taken together, susceptibility of polyisoprenoid alcohols to various oxidizing agents was shown for the first time.

**Electronic supplementary material:**

The online version of this article (doi:10.1007/s11745-015-4104-y) contains supplementary material, which is available to authorized users.

## Introduction

Isoprenoid alcohols are synthesized in all living cells, although their composition and content is species-specific. Plants synthesize both short-chain and long-chain linear (poly)isoprenoid alcohols [1]. The biological function of isoprenoid phosphates is well established; among others, they are utilized as precursors of numerous essential compounds, e.g. carotenoids, tocopherols, chlorophylls and prenylated quinones, while phosphates of long-chain isoprenoid alcohols are cofactors in protein glycosylation [2]. In contrast, the biological role of free isoprenoid alcohols is still the subject of extensive studies. Short-chain isoprenoid alcohols (components of volatile secretion) are thought to be involved in plant response to adverse environmental conditions, pollination attractants, repellents, insect pests or in resisting microbial attack [3]. Interestingly, an antioxidant role was also ascribed to isoprene and geraniol [4, 5]. In line with these data, short-chain isoprenoid alcohols have been shown to act as protectors against abiotic stresses such as high light, elevated temperature or drought [6]. Monoterpenes are currently also widely used as ingredients of various household products, e.g. food flavorings, cosmetics or cleaning preparations [3].

Long-chain isoprenoid alcohols, components all biological membranes, are postulated to modulate their physico-chemical properties (by increasing the fluidity and permeability) [7] and to act as a shield protecting other membrane components against the attack of reactive oxygen species (ROS) [8]. These observations make isoprenoid alcohols important components of the machinery of plant adaptation in response to adverse environmental stimuli. Consequently, upon exposition to ROS, isoprenoid alcohols should be expected to undergo oxidative modifications. Despite their occurrence in all living organisms and their postulated role as ROS scavengers, the products of polyisoprenoid alcohol oxidation by ROS have never been described.

In cells, non-enzymatic lipid peroxidation is conducted mostly by reaction with singlet oxygen [9], and the products formed, as characterized for cholesterol and fatty acids [9, 10], are hydroperoxides (e.g. hydroperoxides of linolenate) epoxides (e.g. 5,6-epoxy-cholesterol) or aldehydes (e.g. malondialdehyde, MDA; 4-hydroxynonenal, 4-HNE).

Numerous investigations on oxidation of short-chain isoprenoids have been performed by various oxidizing agents, e.g. singlet oxygen or molecular oxygen [11].

Singlet oxygen is chemically obtained from photo-catalytic processes or in reactions performed in the absence of light. The latter is achieved by several methods, e.g. the disproportionation reaction of hydrogen peroxide with the use of molybdate ions as a catalyst [12]. This reaction has been used previously for oxidation of citronellol to produce rose oxide via the formation of hydroperoxides and diols [13], and to obtain several other products [14]. Photo-catalytic generation of singlet oxygen is achieved by photosensitization with many substances, such as porphyrins [15], titanium dioxide [16] or rose bengal (RB) [17]. An interesting example is photooxidation of citronellol under visible light in the presence of porphyrins [18].

In parallel, combined systems designed to increase the effectiveness of the oxidation process have been extensively investigated. These so-called advanced oxidation processes (AOPs) are achieved using H_2_O_2_/UV, O_3_/hypochlorite, TiO_2_/UV, O_3_/UV, air/UV, ultrasound (US) and many others [19]. In AOPs, the hydroxyl radical (^**·**^OH), considered as the strongest oxidant, is formed [20]. Nowadays, advanced oxidation processes are often involved in wastewater treatments or in degradation of pesticides [21].

**Fig. 1:**
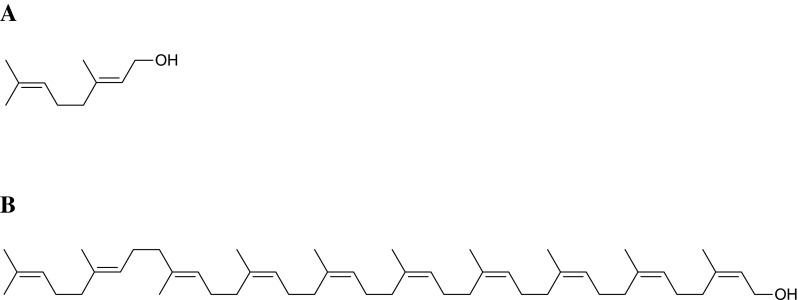
Structure of Prenol-2 (**a**) and Prenol-10 (**b**)

In this report, oxidation of isoprenoid alcohols, Prenol-2 (P-2) and Prenol-10 (P-10) (Fig. 1) by means of several oxidizing agents, i.e. singlet oxygen, hydroxyl radical and molecular oxygen, was investigated. Singlet oxygen was generated in non-photochemical (from hydrogen peroxide in the presence of sodium molybdate) and photo-chemical (in the presence of porphyrin) reactions. Oxidation by hydroxyl radical was performed using the AOP system (in the presence of UV/titanium dioxide, UV/hydrogen peroxide and upon sonication/hydrogen peroxide). Additionally, concentrated (50 %) hydrogen peroxide was also used to generate oxygen upon dismutation process.

In general, both short-chain and long-chain polyisoprenoid alcohols were readily oxidized with formation of mixtures of products. Singlet oxygen caused efficient transformation of P-2 into oxidized (hydroxy, peroxy, oxetane and epoxy) derivatives. In contrast, P-10 was not susceptible to oxidation upon non-photochemical conditions, while upon photo-chemical oxidation, the formation of hydroxy and peroxy derivatives was observed.

In an attempt to establish the structure of oxidized products, electrospray coupled with tandem mass spectrometry (ESI–MS/MS) analysis of selected ions corresponding to the oxidized derivatives of isoprenoid alcohols was performed. Interestingly, products of polyisoprenoid oxidation showed higher affinity to ammonium than lithium cations, recommended thus far for fragmentation analysis of polyisoprenoids and their esters [26]. Results of MS/MS analysis were verified, for Pren-2 derivatives, by nuclear magnetic resonance spectroscopy (NMR).

To the best of our knowledge this is the first attempt to characterize the products formed upon ROS-mediated oxidation of polyisoprenoid alcohols. Practical aspects of these results are discussed.

## Materials and Methods

### Chemicals

Prenol-10 (P-10) was from the Collection of Polyprenols (Institute of Biochemistry and Biophysics, Polish Academy of Sciences, Warsaw). Prenol-2 (P-2; trivial name geraniol), 3-chloroperoxybenzoic acid, sodium molybdate and pyridine *meso*-tetraphenylporphyrin (syn. 5,10,15,20-tetraphenyl-21H,23H porphyrin) and lithium aluminum deuteride were supplied by Sigma-Aldrich, Poznan, Poland. Pyridinium chlorochromate and dimethyl sulfide were purchased from Fluka (Poznan, Poland). Hydrogen peroxide 50 % p.a. was from Chempur (Piekary Slaskie, Poland).

Thin-layer chromatography (TLC) plates (silica gel 60 and RP-18) and silica gel for column chromatography were from Merck (Darmstadt, Germany), Florisil gel (60–100 mesh) was from Sigma-Aldrich (Poznan, Poland). All organic solvents (HPLC and analytical grade) were obtained from POCh (Gliwice, Poland).

### Semi-synthetic Model Compounds

#### Aldehyde Derivatives of Prenol-2 and Prenol-10

Oxidation to aldehydes was performed using methods described earlier [22]. Briefly, isoprenoid alcohol (2.85 mmol of Prenol-2 or 0.054 mmol of Prenol-10) was dissolved in dichloromethane (4 ml) and stirred with pyridinium chlorochromate (4.64 or 0.232 mmol for P-2 or P-10, respectively). The reaction was performed for approximately 20 min at room temperature. Products were purified by two-step flash column chromatography on Florisil gel in dichloromethane, followed by silica gel in hexane with increasing concentrations of diethyl ether (0–15 %) with monitoring by TLC. The slectrospray coupled with mass spectrometry (ESI–MS) and gas chromatography coupled with mass spectrometry (GC/MS) analyses confirmed the structure of the obtained aldehydes.

#### Epoxy Derivatives of Prenol-2 and Prenol-10

Synthesis of epoxy derivatives of isoprenoid alcohols was performed as described previously [23] with some modifications. Briefly, isoprenoid alcohol (19.45 or 0.015 mmol for P-2 or P-10, respectively) was dissolved in dichloromethane (10 ml) and cooled in an ice-water bath. Then a solution of 3-chloroperoxybenzoic acid (40 or 0.0196 mmol for P-2 or P-10, respectively) in dichloromethane (100 ml or 10 ml, respectively) was gradually added to the reaction mixture, and finally, the mixture was stirred for an additional 1 h with cooling. The progress of the reaction was monitored by TLC. The reaction mixture was filtered, evaporated and residual material was dissolved in chloroform/methanol (1:1, by vol). Structures of products were analyzed by high-performance liquid chromatography coupled with ultraviolet absorption detector (HPLC/UV), ESI–MS, HPLC/ESI–MS and GC/MS.

### Oxidation of Prenyl Alcohols with Singlet Oxygen Generated in the Presence of Molybdate

Ten grams (65 mmol) of Prenol-2 and 62.5 ml of ethylene glycol were mixed, then supplemented with an aqueous solution of sodium molybdate (0.7 g; 3.4 mmol) and stirred at 55 °C. Hydrogen peroxide (30 %) was added dropwise to the reaction mixture. Special attention was paid to maintain the molybdate anion (MoO_4_^2−^) concentration in the reaction mixture, which was monitored visually (an orange color of the mixture). Neither tetraperoxomolybdate [Mo(O_2_)_4_]^2−^ nor diperoxomolybdate [MoO_2_(O_2_)_2_]^2−^ (deep—red or yellow color, respectively) are efficient reactants to generate singlet oxygen [13]. The reaction was carried out for 6 h and the progress of reaction was controlled by TLC. Several purification steps were performed to isolate oxidized products of P-2 using column chromatography on silica gel with isocratic elution (ethyl acetate/hexane; 35:65, by vol) under the control of TLC. Three products were isolated and identified with the aid of mass spectrometry (HPLC/ESI–MS and ESI–MS/MS) and NMR spectroscopy (^1^H-, ^13^C NMR).

Numerous attempts to oxidize P-10 with single oxygen using conditions analogous to P-2 were unsuccessful, despite several modifications of the reaction conditions. Thus, various solvent mixtures (2-propanol, ethanol, methanol, ethylene glycol, dimethylformamide, acetonitrile, dichloromethane, ethyl acetate, tetrahydrofuran in different ratios), detergents (sodium lauryl sulphate, Tween 20) and temperatures (20 or 55 °C) were tested with no effect.

### Oxidation of Prenyl Alcohols with Singlet Oxygen Generated in the Presence of Porphyrin

The reaction was performed as described earlier for carbamate derivative [24] with modifications. Briefly, 100 mg (143 µmol) of P-10 or 0.9 g (5.69 mmol) of Prenol-2 was dissolved in 75 ml of the mixture of solvents (2-propanol/ethanol/dichloromethane 1:1:4, by vol or methanol/dichloromethane 1:2, by vol, for P-10 and P-2, respectively) supplemented with 300 µl of pyridine. The reaction was carried out with continuous oxygen bubbling and halogen lamp (200 W) irradiation for 9 h at −78 °C in the presence of porphyrin as a photosensitizer to generate singlet oxygen. Dimethyl sulfite (4 ml) was added to terminate the reaction, and after 10 h the mixture was diluted with hexane and placed on an aluminum oxide column with active carbon as a top layer (to remove porphyrin), and eluted with hexane.

Isolation and structural analysis of the three main products obtained during oxidation of Prenol-2 in the presence of porphyrin was performed as above.

### Reduction of Epoxides with Lithium Aluminum Deuteride

The sample of oxidized P-10 (1.0 mg) was dissolved in a cooled (0 °C) anhydrous solution of lithium aluminum deuteride (0.1 M) in tetrahydrofuran (1 ml). The reaction was carried on in an inert argon atmosphere. The reduction was performed at 0 °C for about 20 min and then the cooling bath was removed and the reaction was continued for 1.5 h at room temperature. The reaction was terminated by the addition of sodium hydroxide solution (0.5 ml, 10 % w/v) and lipids were extracted with THF (15 ml) and subsequently with hexane (15 ml). Organic extracts were pooled and evaporated. Identification of the products was performed using ESI–MS. Reduction of model compound—epoxidized P-10 was performed in parallel.

### Oxidation of Prenyl Alcohol with Oxygen Generated *In Situ* from Hydrogen Peroxide

P-10 (0.6 µmol) was dissolved in 1 ml of 2-propanol/ethanol (1:1, by vol) and then 500 µl of hydrogen peroxide (50 %) was added with mixing. The reaction was continued for 24 h and the progress of the reaction was monitored by mass spectrometry after 1, 3, 5, 12 and 24 h. Samples were extracted three times with 5 ml of ethyl acetate/hexane (4:6, by vol), and after drying with anhydrous sodium sulfate, pooled extracts were evaporated under a stream of nitrogen, dissolved in chloroform/methanol (1:1, by vol) and analyzed by HPLC/ESI–MS and ESI/MS–MS. All the experiments were performed in triplicate.

### Oxidation of Prenyl Alcohols with Hydroxyl Radicals Generated Upon US/H_2_O_2_, UV/H_2_O_2_ or UV/TiO_2_ Treatments (AOPs)

P-2 (0.28 nmol) was dissolved in 500 µl of ethanol and P-10 (0.6 µmol) in 200 µl of 2-propanol/ethanol (1:1, by vol),

*US/H*_*2*_*O*_*2*_ prenol solutions were mixed with 500 µl of hydrogen peroxide (50 %) and sonicated (Sonics VC 505, Sonics & Materials Inc., USA) for 5 or 30 min for P-2 or P-10, respectively. The amplitude of sonication was set at 40 %.

*UV/H*_*2*_*O*_*2*_ prenol solutions were mixed with hydrogen peroxide (50 %, 200 µl) and exposed to irradiation by a UV lamp (NBV 2 × 30 V, ULTRA VIOL UV).

*UV/TiO*_*2*_ prenol solutions, after addition of titanium dioxide (10 mg), were exposed to UV irradiation as above.

Control samples—prenol solutions—were exposed to UV irradiation.

For all AOP procedures, the reaction was continued for 0.5, 1, 3, 5 or 15 h. The progress of the reaction was monitored by TLC and HPLC/UV. The reaction mixture was extracted as above, and the products were analyzed by HPLC/ESI–MS and ESI–MS/MS, and additionally by GC/MS for Prenol-2. All the experiments were performed in triplicate.

### TLC Analysis

Progress of the reactions was monitored by TLC. Silica gel and RP-18 plates were used. Silica gel plates were developed in mixture of hexane and diethyl acetate (1:1, or 3:1, by vol for P-2 or P-1-, respectively) and visualized with iodine vapors, or alternatively by spraying the plate with either cerium sulfate (IV) spray (aqueous solution of 10 % cerium (IV) sulfate and 15 % H_2_SO_4_) followed by heating with hot-air. RP-18 plates were developed in acetone or in a mixture of acetone/methanol (95:5, by vol) and exposed to iodine vapors.

### HPLC/UV Analysis

The analysis of short and long-chain isoprenoids and their oxidation products were performed according to a previously described protocol [25] with modifications. Analyses were performed using a 4.6 × 75 mm ZORBAX XDB-C18 (3.5 µm) reversed-phase column (Agilent, USA) using a Waters 515 dual-pump apparatus. Waters gradient programmer and a Waters 2996 Photodiode Array Detector (spectrum range 210–400 nm) were used.

For elution of short-chain isoprenoids and their derivatives, a combination of two phases was used: solvent A was methanol/water (2:8, by vol), and solvent B was methanol/water (9:1, by vol). A linear gradient was used: for the initial 4 min, 100 % of phase A; from 100 to 40 % phase A for the next 8 min; 40 % phase A for the next 4 min; from 40 to 20 % phase A during the following 2 min; then from 20 to 100 % of A for 2 min, and in the last 3 min, re-equilibration back to 100 % A was performed. The solvent flow rate was 1.3 ml/min.

In the case of P-10, the phases were applied as previously described [25] and the linear gradient was as follows: 100 % of phase A for 2 min; from 100 to 85 % A for the next 8 min; from 85 to 60 % phase A during the next 25 min; then from 60 to 55 % of phase A for 5 min, and in the last 2 min, re-equilibration back to 100 % A. The solvent flow rate was 1.5 ml/min. The identity and quantitative determination of isoprenoids was confirmed by applying qualitative external standards (P-2, P-10). Integration of the HPLC/UV chromatograms was performed with the aid of Empower (Waters) software.

### HPLC/ESI–MS and ESI–MS/MS Analysis

The samples were dissolved in a mixture of methanol/2-propanol (1:1; by vol) or chloroform/methanol (1:1, by vol) and injected into the HPLC (Prominence LC-20; Shimadzu). Chromatographic conditions were as above.

MS spectra were recorded on a mass spectrometer 4000 Q TRAP (Applied Biosystems Inc, USA) equipped with an electrospray (ESI) ion source (TurboIonSpray) and the triple quadrupole/linear ion trap mass analyzer. The instrument was controlled and recorded data were processed using the *Analyst v. 1.4.2* software package (Applied Biosystems Inc, USA). The ESI-MS spectra were obtained with and without salt addition in the positive ion mode, in the *m*/*z* range 100–1000 or 100–2000, depending on the investigated compounds. The ion source parameters were optimized to obtain the best intensity of the investigated peaks.

MS/MS analysis (CID spectra) of selected ions was performed in the positive ion mode. Variable collision energy (CE) (10–60 eV) was applied depending on the product structure, as indicated in the results. Nitrogen was employed as a collision gas, as well as a nebulizer and a curtain gas. As indicated, ammonium or lithium acetate was added to the analyzed mixture prior to injection.

### GC/FID and GC/MS Analysis

These methods were used exclusively for oxidized derivatives of short-chain isoprenoids.

The GC apparatus (Agilent Technologies, 7890A) was equipped with a split/splitless injector and a flame ionization detector (FID). A capillary HP-5 column (J & W Scientific Columns for Agilent Technologies) of 30 m length, 0.32 mm internal diameter and 0.25 µm film thickness was used. Nitrogen, hydrogen and air flow-rates were maintained at 15, 30 and 400 ml/min, respectively. The inlet and detector temperature was kept at 250 and 300 °C, respectively, and the oven temperature was programmed as 100 °C with a 5-min hold at 100 °C, an increase rate of 10 °C/min to 290 °C and a 10-min hold at 290 °C.

Gas chromatography–mass spectrometry analyses were performed on a 7890A gas chromatograph (Agilent Technologies) coupled with a 5975C single quadrupole mass spectrometer (Agilent Technologies) equipped with a triple-axis detector and an electron ionization ion source. The GC/MS apparatus was equipped with a split/splitless injector. A capillary HP-5 ms column 30 m × 0.25 mm, 0.25 μm (J & W Scientific Columns for Agilent Technologies) was used. Helium was used as a carrier gas at a flow rate of 1 ml/min in the constant flow mode and the air flow-rate was maintained at 400 ml/min. The inlet and detector temperature was kept at the same temperature as above and also the oven temperature was programmed in the same way. Full-scan electron impact (EI) ionization spectra were recorded in the range of *m*/*z* 35–650. Preliminary oxidized compound identifications from the Agilent 7890A/5975C GC/MS data sets were made by comparing their spectra with those collected in the NIST Mass Spectral Program (NIST/EPA/NIH Mass Spectral Library Version 2.0f, build Oct 8, 2008).

### NMR Analysis of Prenol-2 Oxidized Products

For NMR measurements, samples were dissolved in deuterochloroform (CDCl_3_). Spectra were measured on the Varian VNMRS 600 MHz spectrometer at 25 °C. One-dimensional (1-D) ^1^H and ^13^C spectra and 2-D ^1^H-^1^H COSY, ^1^H-^13^C HSQC, and ^1^H-^13^C HMBC spectra were acquired. Additionally, 1-D NOESY spectra have been recorded for the selected proton signals. Plots of the original spectra prepared using Spinworks v. 4.05 software are shown in the Supplemental Figure 1.

Obtained data are summarized below.

### Products Obtained Under “Dark” Conditions

#### 2-(Hydroxymethyl)-6-Isopropenyl-3-Methyl-Tetrahydropyran-3-Ol

^1^H NMR: 4.95 (d, 2H), 4.45 (dd, 1H), 4.25 (dd, 1H), 3.95 (dd, 1H), 3.65 (m,1H), 2.76 (s, 1H), 2.26 (m, 1H), 1.9 (m,1H), 1.74 (m, 5H), 1.59 (m, 1H), 1.11 (s, 3H); ^13^C NMR: 142.37 (C1), 113.43 (C2), 83.21 (C3), 80.84 (C4), 72.03 (C5), 62.72 (C6), 30.12 (C7), 24.00 (C8), 20.21 (C9), 19.59 (C10);

#### 1-(5-Isopropenyl-2-Methyl-Tetrahydrofuran-2-Yl)Ethane-1,2-Diol

^1^H NMR: 5.00 (s, 1H); 4.83 (s, 1H), 4.38 (t, 1H), 3.77 (m, 1H), 3.70 (m, 1H), 3.61 (m, 1H), 2.12 (m, 2H), 1.74 (m, 5H), 1.62 (m, 1H), 1.24 (m, 4H); ^13^C NMR: 144.87 (C1), 110.65 (C2), 84.65 (C3), 80.82 (C4), 76.63 (C5), 63.18 (C6), 33.09 (C7), 31.25 (C8), 22.61 (C9), 18.24 (C10);

#### 3-[-4-Hydroperoxy-4-Methyl-Pent-2-Enyl]-3-Methyl-Oxiran-2-Yl]-Methanol (Main Component)

^1^H NMR: 8.2 (s, 1H), 5.63 (m, 2H), 3.81 (m, 1H), 3.70 (m, 1H), 3.02 (m, 1H), 2.32 (m, 2H), 1.32 (m, 10H); ^13^C NMR: 137.69 (C1), 125.29 (C2), 81.91 (C3), 62.07 (C4), 61.29 (C5), 60.99 (C6), 40.91 (C7), 24.24 (C8), 24.09 (C9), 17.11 (C10).

### Products Obtained Under “Light” Conditions

#### (2*E*)-3,7-Dimethylocta-2,7-Diene-1,6-Diol

^1^H NMR (CDCl_3_): 1.62–1.72 (m, 2H, H5, H5′), 1.69 (s, 3H, H10), 1.73 (s, 3H, H9), 1.97 (bs, 2H, 2 × OH), 2.01–2.13 (m, 2H, H4, H4′), 4.05 (m, 1H, H6), 4.15 (d, 2H, *J* = 6.9 Hz, H1), 4.85 (m, 1H, H8), 4.94 (m, 1H, H8′), 5.44 (m, 1H, H2). ^13^C NMR (CDCl_3_): 16.2 (C10), 17.5 (C9), 32.8 (C5), 35.4 (C4), 59.2 (C1), 75.4 (C6), 111.1 (C8), 123.6 (C2), 139.2 (C3), 147.4 (C7).

#### (2*E*,5*E*)-3,7-Dimethylocta-2,5-Diene-1,7-Diol

^1^H NMR (CDCl_3_): 1.32 (s, 6H, H8, H9), 1.66 (s, 3H, H10), 1.88 (bs, 2H, 2 x OH), 2.71 (d, 2H, *J* = 6.4 Hz, H4), 4.15 (dd, 2H, *J* = 6.9, 0.6 Hz, H1), 5.42 (m, 1H, H2), 5.60–5.66 (m, 2 H, H5, H6). ^13^C NMR (CDCl_3_), 16.3 (C10), 29.7 (C8, C9), 42.1 (C4), 59.2 (C1), 70.6 (C7), 124.2 (C2), 124.3 (C5), 138.1 (C3), 140.0 (C6).

#### (2*E*,5*E*)-7-Hydroperoxy-3,7-Dimethylocta-2,5-Dien-1-Ol

^1^H NMR (CDCl_3_): 1.34 (s, 6H, H8, H9), 1.67 (s, 3H, H10), 1.96 (bs, 1H, OH), 2.75 (d, 2H, *J* = 6.4 Hz, H4), 4.16 (d, 2H, *J* = 6.9 Hz, H1), 5.43 (m, 1H, H2), 5.59–5.70 (m, 2H, H5, H6), 8.13 (bs, 1H, –OOH). ^13^C NMR (CDCl_3_): 16.4 (C10), 24.3 (C8, C9), 42.3 (C4), 59.3 (C1), 82.0 (C7), 124.2 (C2), 128.6 (C5), 135.6 (C6), 138.1 (C3).

## Results

### Structural Elucidations of Model Compounds: Prenol-2 and Prenol-10 and Their Oxidized Derivatives

Chemical syntheses of oxidized prenyl alcohols—aldehydes and oxides—of Prenol-2 and Prenol-10 (Fig. 1) were performed using well established methods. As expected, aldehydes or oxides were formed during oxidation of the respective alcohol (P-2 or P-10) with pyridinium chlorochromate or 3-chloro-peroxybenzoic acid, respectively. Their structures were confirmed using HPLC/ESI–MS and ESI–MS/MS. Ammonium or lithium acetate was added to improve the fragmentation process by an ammoniated or lithiated parent ion formation, since sodiated ions of none of the analyzed compounds, i.e. P-2, P-10 and their derivatives, were prone to fragmentation at any conditions tested. In contrast, fragmentation of ammoniated and lithiated ions revealed specific fragmentation patterns, and this phenomenon was in accordance with earlier report showing fragmentation of the lithiated prenyl ions [26]. Fragmentation patterns thus recorded were further used for structural elucidations of the new oxidized P-2 and P-10 derivatives.

### Structural Elucidations of Model Compounds: Prenol-2 Aldehyde and Epoxides

The ESI–MS spectra of Prenol-2 and its oxides were dominated by sodiated signals of investigated compounds. The fragmentation spectra were performed for each of the Prenol-2 derivatives. The fragmentation (collision-induced dissociation; CID) spectrum of the substrate—Prenol-2 was performed with ammonium acetate addition. In this spectrum the loss of water and a 28 Da fragment, probably corresponding to an ethene molecule, was observed (*m*/*z* 137.0, 109.0).

Results of MS/MS analysis of oxidized Prenol-2 derivatives are provided in the Supplemental Table 1 and accompanying comments.

In summary, upon MS/MS experiments (CE 10 eV), fragmentation of Prenol-2 or Prenal-2 resulted in the loss of one water molecule, while for monoepoxide and diepoxide, it resulted in the loss of two or three water molecules, respectively (Supplemental Table 1).

### Structural Elucidations of Model Compounds: Prenol-10 Aldehyde and Epoxides

The fragmentation (CID) spectrum of Prenol-10 ([M + Li]^+^*m*/*z* 705.6) revealed the presence of *m*/*z* 687.5 and *m*/*z* 619.5 ions corresponding to the loss of one water molecule and one isoprene residue (C_5_H_8_). Moreover, in the range of *m*/*z* 143.1–551.5 low intensity signals were observed, with *m*/*z* differing by 68 amu, thus corresponding to the loss of the subsequent isoprene residues (Supplemental Table 2). This fragmentation pathway is in accordance with the fragmentation pattern noted previously for P-8, 9, 10, 11 and 12 [26].

For Prenal-10 the sodiated ion at *m*/*z* 719.8 was recorded. The fragmentation pattern of the ammoniated ion *m*/*z* 714.9 analogously as for Prenal-2, indicates the loss of one water molecule (*m*/*z* 679.8) and elimination of an ethene molecule (*m*/*z* 651.8) (Supplemental Table 2).

Epoxidation of P-10 resulted in formation of a complex mixture of oxides, which, after a HPLC–UV run, was separated into ten fractions (respective ‘time windows’, Supplemental Table 3). In ESI–MS and HPLC/ESI–MS analyses of the polyepoxide mixture (Fraction1), monoepoxides (Fractions No 2–10), traces of diepoxides (Fractions No 2–6) and unreacted P-10 were identified. In ESI–MS spectra of all oxides, recorded by direct MS analysis and HPLC/ESI–MS as well, sodiated ions of the investigated compounds were obtained (see Supplemental Table 3). Our previous studies showed that sodiated ions of prenyl alcohols were not susceptible to fragmentation; thus, the sample was supplemented with lithium acetate prior to the ESI–MS/MS analysis [26]. As expected, lithiated ions corresponding to P-10 (*m*/*z* 705.6), P-10 monoepoxide (*m*/*z* 721.6) and oligoepoxides up to hexaepoxide (*m*/*z* 801.9) were found (Supplemental Table 3).

MS/MS analysis of selected P-10 epoxides was performed with lithium or ammonium salt addition to establish their structures.

Results of MS/MS analysis of oxidized Prenol-10 derivatives are provided in the Supplemental Table 2 and accompanying comments.

In general, the fragmentation pattern of P-10 epoxides (CE 30 eV) indicates that the loss of two water molecules is specific for monoepoxides of both short-chain and long-chain polyisoprenoids, while the loss of three water molecules is characteristic for diepoxides, tri- and polyepoxides of P-10. The fragmentation pathway for lithiated and ammoniated ions of the investigated compounds seems to occur in two steps: in the first step, there is the loss of water molecules, and in the next, the elimination of isoprene residues, reflecting the length of the analyzed polyprenol.

### Oxidation of Prenyl Alcohols, Prenol-2 and Prenol-10, with Singlet Oxygen Generated in the Presence of Molybdate

In a first line of experiments, singlet oxygen was generated from hydrogen peroxide in the presence of molybdate salt [13]. Upon these so-called ‘dark’ conditions, oxidation of Prenol-2 revealed three main oxidized products (Table 1). HPLC/ESI–MS analysis of the reaction mixture showed two signals at *m*/*z* 209.4 and one at *m*/*z* 225.3. The reaction mixture was separated by several successive chromatographic runs on silica gel columns (see “Materials and methods” section) to obtain pure products. After isolation and purification, structural analysis (HPLC/ESI–MS, ESI–MS/MS and NMR) was performed separately for each product (Product_P-2_ No 1–3).

**Table 1:** Products of oxidation of Pren-2 with singlet oxygen generated in the presence of sodium molybdate and hydrogen peroxide—mass spectrometry (ESI–MS and ESI–MS/MS) and NMR analyses

Products of Pren-2	Molecular ion *m*/*z*		MS/MS analysis—daughter ions		Postulated structure
	[M + Na]^+^	[M + NH_4_]^+^	*m*/*z*	Fragmentation path	
Product_Pren-2_ No 1 (P-2-1)	209.4		N.D.	N.D.	2-(Hydroxymethyl)-6-isopropenyl-3-methyl-tetrahydropyran-3-ol
		204.1	169.0 151.1 133.0 105.0	[M_P-2-1_ + NH_4_ − NH_3_ − H_2_O]^+^ [M_P-2-1_ + NH_4_ − NH_3_ − 2H_2_O]^+^ [M_P-2-1_ + NH_4_ − NH_3_ − 3H_2_O]^+^ [M_P-2-1_ + NH_4_ − NH_3_ − 3H_2_O − 28 Da]^+^	
Product_Pren-2_ No 2 (P-2-2)	209.4		N.D.	N.D.	1-(5-Isopropenyl-2-methyl-tetrahydrofuran-2-yl)ethane-1,2-diol
		204.1	169.1 151.1 133.0	[M_P-2-2_ + NH_4_ − NH_3_ − H_2_O]^+^ [M_P-2-2_ + NH_4_ − NH_3_ − 2H_2_O]^+^ [M_P-2-2_ + NH_4_ − NH_3_ − 3H_2_O]^+^	
Product_Pren-2_ No 3 (P-2-3)	225.3		N.D.	N.D.	3-[-4-Hydroperoxy-4-methyl-pent-2-enyl]-3-methyl-oxiran-2-yl-methanol
		220.0	185.1 169.1 151.0 133.0	[M_P-2-3_ + NH_4_ − NH_3_ − H_2_O]^+^ [M_P-2-3_ + NH_4_ − NH_3_ − H_2_O − 16 Da]^+^ [M_P-2-3_ + NH_4_ − NH_3_ − 2H_2_O − 16 Da]^+^ [M_P-2-3_ + NH_4_ − NH_3_ − 3H_2_O − 16 Da]^+^	

Ammoniated adducts [M + NH_4_] were subjected to fragmentation analysis

In the ESI–MS spectrum of Product_P-2_ No 1 (P-2-1) the sodiated ion at *m*/*z* 209.4 indicated the possible presence of two additional oxygen atoms in the Prenol-2 molecule (Table 1). After supplementation with ammonium acetate, the fragmentation spectrum of the respective ammoniated molecular ion at *m*/*z* 204.1 revealed the following fragmentation pattern: loss of three subsequent water molecules (*m*/*z* 169.0, 151.1, 133.0) and probably an ethene fragment (*m*/*z* 105.0) (Table 1). Such a fragmentation pattern might suggest that Product_P-2_ No 1 was a diepoxide, but the necessity of applying collision energy of 40 eV, which is significantly higher than 10 eV (for fragmentation of P-2 diepoxide 10 eV was required) caused us to question this supposition and suggested a different structure of Product_P-2_ No 1 than diepoxide. Detailed analysis of the ^1^H and ^13^C NMR spectra, as well as 2-D NMR ^1^H–^1^H and ^1^H–^13^C correlations together with MS/MS analysis revealed the structure of Product_P-2_ No 1 as 2-(hydroxymethyl)-6-isopropenyl-3-methyl-tetrahydropyran-3-ol (Table 1; “Materials and methods” section NMR analysis). It was clearly visible that the molecule of compound P-2-1 contains isopropenyl group, one methyl group and two hydroxyl groups. The molecular formula C_10_H_18_O_3_ indicates two double bond equivalents, so consequently, the third oxygen atom must be a part of the ring (no carbonyl group is present according to ^13^C NMR spectrum). Further analysis of the H–H and C–H couplings finally led to the tentative structure P-2-1. The compound of this structure has not yet been described in the literature, but fortunately, its structure has been supported by the structure of the strictly related product P-2-2, which appeared to be known (see below).

**Fig. 2:**
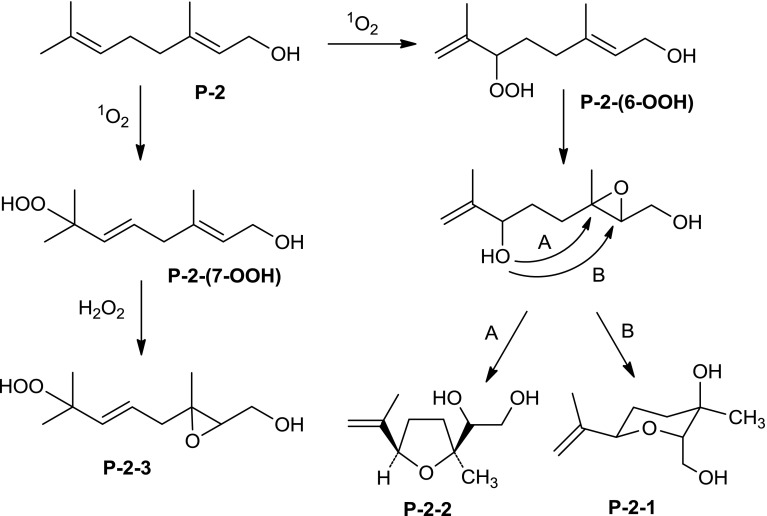
Oxidation of Prenol-2 with hydrogen peroxide and singlet oxygen generated in the ‘dark’ conditions (in presence of sodium molybdate)—proposed pathways leading to the products P-2-1, P-2-2, and P-2-3

In the ESI–MS spectrum of the second oxidized product—Product_P-2_ No 2 (P-2-2), sodiated molecular ion at *m*/*z* 209.4 was observed, which suggested the presence of three additional oxygen atoms in its molecule (C_10_H_18_O_3_). Fragmentation of the ammoniated ion (*m*/*z* 204.1) showed the elimination of ammonium and water molecule (*m*/*z* 169.1) and then the loss of two water molecules (*m*/*z* 151.1 and 133.0) (Table 1). Based on the fragmentation path and NMR analyses, as described for the product P-2-1, the structure of Product_P-2_ No 2 was suggested as 1-(5-isopropenyl-2-methyl-tetrahydrofuran-2-yl)-ethane-1,2-diol (Table 1; “Materials and methods” section NMR analysis). In this case the literature search for compounds with this or a similar structure was successful. In 2003 Marshall and Chobanian described the synthesis of compound with the NMR spectrum identical within experimental error with the spectrum of the product P-2-2. [27]. This finding confirms unequivocally not only the structure of compound P-2-2, but also supports strongly the structure of the P-2-1 product, because these two compounds are most likely formed from the same precursor (see Fig. 2; “Discussion” section below).

For Product_P-2_ No 3 (P-2-3) sodiated ion at *m*/*z* 225.3 was recorded, while in the CID spectrum of the ammoniated ion (*m*/*z* 220.0), there was the loss of the following fragments: an ammonium and water molecule (*m*/*z* 185.1) then a 16 Da fragment (*m*/*z* 169.1) and two sequential water molecules (*m*/*z* 151.0 and 133.0, respectively) (Table 1). According to these results, the molecular formula C_10_H_18_O_4_ has been proposed. MS/MS and NMR analysis suggested the structure of Product_P-2_ No 3 as 3-[-4-hydroperoxy-4-methyl-pent-2-enyl]-3-methyl-oxiran-2-yl]-methanol (Table 1; “Materials and methods” section NMR analysis). From the NMR spectra, it was clear that the molecule—in contrast to the products P-2-1 and P-2-2—contains three methyl groups and a hydroperoxyl group. Detailed analysis of the 1-D and 2-D spectra led to the proposed formula. It has to be noted, however, that almost all signals in ^1^H and ^13^C NMR spectra of P-2-3 are split into two components, indicating that this product is a mixture of two diastereoisomers, differing only slightly by their structures. It was not possible to assign their stereochemical structures on the basis of the NMR experiments performed. We also were not able to find the compound with this or similar structure in the literature.

Taken together, three main products—hydroxy, peroxy and heterocyclic derivatives—were obtained during oxidation of Prenol-2 with singlet oxygen generated in the H_2_O_2_/sodium molybdate system. Putative degradation pathways of Prenol-2 are shown in Fig. 2.

In the first step, two possible ene-type reaction products of P-2 with singlet oxygen leading to the hydroperoxides P-2-(6-OOH) and P-2-(7-OOH) are formed (Fig. 2). The formation of such products has been described by Sels *et al*. [28]. The first product can undergo transformation to the hydroxy epoxide, which can be opened intramolecularly, yielding tetrahydropyran derivative P-2-1, and tetrahydrofuran one (P-2-2). P-2-(7-OOH) can be epoxidized by H_2_O_2_, finally yielding product P-2-3. In this case, the order of reactions can be reversed, i.e. the epoxidation reaction can precede the *ene*-type addition of singlet oxygen. It has to be noted that the intermediate P-2-(7-OOH) cannot undergo reactions similar to P-2-(6-OOH) due to the steric reasons (the presence of C5-C6-double bond).

Parallel treatment of Pren-10 with singlet oxygen using reaction conditions described above did not result in formation of detectable amount of products. The yield of P-10 oxidation was negligible, most probably due to much higher hydrophobicity of P-10 then P-2 (C-50 and C-10 alcohol, respectively). Several experimental conditions were tested in order to achieve oxidation of P-10 with singlet oxygen in H_2_O_2_/sodium molybdate system; however, neither the supplementation of the reaction mixture with various organic solvents nor ethylene glycol (see “Materials and methods” section), used previously to enhance the rate of oxidation of short-chain isoprenoids [29] were effective (data not shown).

### Oxidation of Prenyl Alcohols, Prenol-2 and Prenol-10, with Singlet Oxygen Generated in the Presence of Porphyrin

In the second line of experiments, singlet oxygen was generated during irradiation of the reaction mixture with UV light in the presence of porphyrin as a photosensitizer (‘light’ conditions) [24].

Oxidation of Prenol-2 resulted in formation of three main products, which were chromatographically purified and identified by HPLC/ESI–MS and NMR. In the ESI–MS spectra, sodiated and ammoniated ions were present. In all cases, *m*/*z* suggested formation of oxidized products (Table 2). In the next step, ammoniated ions (*m*/*z* 188.0, 188.2 and 204.2) were subjected to MS/MS analysis (CE 30–40 eV) to elucidate their structure.

**Table 2:** Products of oxidation of Pren-2 with singlet oxygen generated in the presence of porphyrin (‘light’ conditions, L)—mass spectrometry analysis (ESI–MS and ESI–MS/MS) and NMR analyses

Products of Pren-2	Molecular ion *m*/*z*		MS/MS analysis—daughter ions		Postulated structure
	[M_P_ + Na]^+^	[M_P_ + NH_4_]^+^	*m*/*z*	Fragmentation path	
Product_P-2_ No 1L (P-2-1L)	193.4		N.D. 171.2 153.3 135.3 107.1	N.D. [M_P-2-1L_ + NH_4_ − NH_3_]^+^ [M_P-2-1L_ + NH_4_ − NH_3_ − H_2_O]^+^ [M_P-2-1L_ + NH_4_ − NH_3_ − 2H_2_O]^+^ [M_P-2-1L_ + NH_4_ − NH_3_ − 2H_2_O − 28 Da]^+^	(2*E*)-3,7-Dimethylocta-2,7-diene-1,6-diol
		188.0			
Product_P-2_ No 2L (P-2-2L)	193.4		N.D. 170.1 153.2 135.3 109.2 107.2	N.D. [M_P-2-2L_ + NH_4_ − H_2_O]^+^ [M_P-2-2L_ + NH_4_ − H_2_O − NH_3_]^+^ [M_P-2-2L_ + NH_4_ − H_2_O − NH_3_ − H_2_O]^+^ [M_P-2-2L_ + NH_4_ − H_2_O − NH_3_ − H_2_O − 26 Da]^+^ [M_P-2-2L_ + NH_4_ − NH_3_ − 2H_2_O − 28 Da]^+^	(2*E*,5*E*)-3,7-Dimethylocta-2,5-diene-1,7-diol
		188.2			
Product_P-2_ No 3L (P-2-3L)	209.3		N.D. 187.2 169.1 153.3 135.3 151.0 133.3 123.2	N.D. [M_P-2-3L_ + NH_4_ − NH_3_]^+^ [M_P-2-3L_ + NH_4_ − NH_3_ − H_2_O]^+^ Path A [M_P-2-3L_ + NH_4_ − NH_3_ − H_2_O − O]^+^ [M_P-2-3L_ + NH_4_ − NH_3_ − H_2_O − O − H_2_O]^+^ Path B [M_P-2-3L_ + NH_4_ − NH_3_ − 2H_2_O]^+^ [M_P-2-3L_ + NH_4_ − NH_3_ − 3H_2_O]^+^ [M_P-2-3L_ + NH_4_ − NH_3_ − 2H_2_O − 28 Da]^+^	(2*E*,5*E*)-7-Hydroperoxy-3,7-dimethylocta-2,5-dien-1-ol
		204.2			

Ammoniated adducts [M + NH_4_] were subjected to fragmentation analysis

Comparison of the fragmentation spectra of ammoniated ions of Prenol-2 oxidation products - Product_P-2_ No 1L, (P-2-1L, ion at *m*/*z* 188.0) and the standard of Prenol-2 monoepoxide (Supplemental Table 1) indicated a similar fragmentation path (Table 2). NMR analysis of the structure of P-2-1L revealed, however, the presence of two hydroxyl groups rather than an epoxy ring in this product (Table 2; “Materials and methods” section NMR analysis). Based on NMR spectra, P-2-1L was identified as (2*E*)-3,7-dimethylocta-2,7-diene-1,6-diol. Interestingly, this compound was earlier identified as a component of rose flowers [30], compound No 6.

The second product of oxidation of Prenol-2 upon light conditions, Product_P-2_ No 2L (P-2-2L) displayed the same molecular mass as P-2-1L, i.e. *m*/*z* 188.2 for ammoniated ion. Despite this fact, fragmentation paths of these two products were slightly different; namely, the sequence of the loss of water and ammonia molecules was different (Table 2). Interestingly, NMR spectra revealed that P-2-1L and P-2-2L are isomeric diols (Table 2; “Materials and methods” section NMR analysis), since P-2-2L was identified as (2*E*,5*E*)-3,7-dimethylocta-2,5-diene-1,7-diol. P-2-2L, similarly to P-2-1L, was also identified as a component of rose flowers [30], compound No 5.

MS analysis of the third product, Product_P-2_ No 3L (P-2-3L, *m*/*z* 204.2 for ammoniated ion) might suggest the presence of three oxygen atoms in its molecule. In the CID spectrum of P-2-3L (Table 2), signals corresponding to loss of ammonia (*m*/*z* 187.2) followed by loss of three subsequent water molecules (*m*/*z* 169.1, 151.0, 133.3) was observed. An alternative path—loss of ammonia followed by loss of one water molecule, then a 16-Da fragment (possibly oxygen) and a second water molecule (*m*/*z* 153.3, 135.3) was observed too. This pointed to the fact that P-2-3L differed structurally from the two products mentioned earlier, namely P-2-1L or P-2-2L. Consistent with this observation, NMR spectra permitted identification of P-2-3L as (2*E*,5*E*)-7-hydroperoxy-3,7-dimethylocta-2,5-dien-1-ol (Table 2; “Materials and methods” section NMR analysis). NMR spectrum of P-2-3L resembled that of P-2-2L, while the presence of the additional oxygen atom was indicated by different chemical shift of the –OOH proton (8.1 ppm), compared to the chemical shift of the –OH proton (about 2 ppm) and MS spectrum. P-2-3L was obtained earlier as a product (No 4a) of photo-oxygenation of geraniol [31], although its structure has not yet been described.

Taken together, three main products—two isomeric hydroxy derivative and one peroxy derivative—were obtained during photochemical oxidation of Prenol-2 with singlet oxygen.

The *m*/*z* values for products of geraniol oxidation obtained in the ‘light’ and ‘dark’ conditions clearly indicate formation of molecules containing additional oxygen atoms, although the profile of products was different. This is indicated by the differences in the *m*/*z* of the molecular ions, as well as their fragmentation paths and NMR spectra (Tables 1, 2).

P-10 was subjected to oxidation with singlet oxygen similarly to P-2. The complex mixture of products was chromatographically purified and products were analyzed by ESI–MS (Supplemental Figure 2) to establish their structures. The main products formed during the reaction with singlet oxygen generated in the presence of porphyrin or formed in upon UV treatment are represented by signals with *m*/*z* corresponding to the sequential increase of the ion mass *Δm*/*z* 16 (Product_P-10_ No 1L–10L), which indicates the presence of from nine to 19 oxygen atoms in oxidized P-10 molecules (Table 3). Subsequent MS/MS analysis of the three most prominent parent ions formed during oxidation of Pren-10 was performed after addition of ammonium acetate (Table 4). The CID spectra (recorded at CE 40–60 eV) showed the loss of three subsequent water molecules for each of these ions *m*/*z* 907.0, 849.5, 831.5; *m*/*z* 922.5, 904.5, 886.5 and *m*/*z* 938.5, 920.5, 902.5 for *m*/*z* 925.5, 940.4 and 956.5, respectively. The observed fragmentation pattern (elimination of three water molecules) might suggest the possible presence of the epoxides in these highly oxidized P-10 molecules. To verify the presence of the epoxide rings, reduction using lithium aluminum deuteride was performed. The ESI–MS analysis of the thus-obtained reduced products showed the main ion at *m*/*z* 963.6, which was not present in the initial mixture after oxidation. Moreover, ions *m*/*z* > 897.8 were not visible after reduction, and neither were the signals corresponding to hydroxylated P-10 molecules, which should have appeared upon the reduction of putative epoxides. In contrast, reduction of the model compound epoxidized P-10 resulted in formation of hydroxylated P-10 derivatives, typical for reduction of epoxides (*m*/*z* 739.7, 755.9, 771.9). Such a profile of reduced products indicates that the substrates of reduction, i.e. products of P-10 photo-oxidation with singlet oxygen, although prone to reduction were not epoxides. Simultaneously, the signals corresponding to the oxidized products of Prenol-10 containing nine and ten oxygen atoms (*m*/*z* 865.7 and 881.9; Table 4) were preserved in the spectrum after treatment with lithium aluminum deuteride (data not shown). This observation strongly suggested that these oxidized Prenol-10 derivatives were not susceptible to reduction. Consequently, P-10 derivatives containing <10 oxygen atoms in the molecule most probably contain hydroxy-groups.

**Table 3:** Selected oxidized products of Pren-10 formed upon reaction with singlet oxygen generated in the presence of porphyrin—mass spectrometry analysis (ESI–MS and ESI–MS/MS)

Product	Products of Pren-10 *m*/*z*	Number of additional oxygen atoms enriched in Prenol-10 molecule [M_Prenol-10_ + n *x* O + Na]^+^
*Singlet oxygen treatment*		
Product_P-10_ No 1L	865.7	9
Product_P-10_ No 2L	881.8	10
Product_P-10_ No 3L	897.8	11
Product_P-10_ No 4L	913.8	12
Product_P-10_ No 5L	929.9	13
Product_P-10_ No 6L	945.8	14
Product_P-10_ No 7L	961.8	15
Product_P-10_ No 8L	977.8	16
Product_P-10_ No 9L	993.8	17
Product_P-10_ No 10L	1009.8	18
Product_P-10_ No 11L	1025.8	19
*UV treatment*		
Product_P-10_ No 12L	737.8	1
Product_P-10_ No 13L	753.8	2
Product_P-10_ No 14L	769.8	3
Product_P-10_ No 15L	785.8	4
Product_P-10_ No 16L	801.8	5
Product_P-10_ No 17L	817.8	6
Product_P-10_ No 18L	833.8	7
Product_P-10_ No 19L	849.8	8
Product_P-10_ No 20L	865.8	9
Product_P-10_ No 21L	881.9	10
Product_P-10_ No 22L	897.9	11
Product_P-10_ No 23L	913.9	12

Ammoniated adducts [M + NH_4_] were subjected to fragmentation analysis

**Table 4:** Products of oxidation of Pren-10 (MW 698.6) after hydrogen peroxide treatment at 55 °C after 1 h—mass spectrometry analysis (ESI–MS and ESI–MS/MS)

Products of Pren-10	Molecular ion *m*/*z*		MS/MS analysis—daughter ions	
	[M_P_ + Na]^+^	[M_P_ + NH_4_]^+^	*m*/*z*	fragmentation path
Product_P-10_ No 5L	929.9		N.D.	N.D.
				Path A
		924.5	908.0 892.0	[M + NH_4_ − NH_3_]^+^ [M + NH_4_ − NH_3_ − 16 Da]^+^
				Path B
			907.0	[M + NH_4_ − NH_3_ − H_2_O]^+^
				Path C
			849.5 831.5	[M + NH_4_ − NH_3_ − 2H_2_O]^+^ [M + NH_4_ − NH_3_ − 3H_2_O]^+^
Product_P-10_ No 6L	945.8		N.D.	N.D.
				Path A
		940.4	923.4 907.4	[M + NH_4_ − NH_3_]^+^ [M + NH_4_ − NH_3_ − 16 Da]^+^
				Path B
			922.5 904.5 886.5	[M + NH_4_ − H_2_O]^+^ [M + NH_4_ − 2H_2_O]^+^ [M + NH_4_ − 3H_2_O]^+^
				Path C
			843.6 825.5	[M + NH_4_ − 2H_2_O]^+^ [M + NH_4_ − 3H_2_O]^+^
Product_P-10_ No 7L	961.8		N.D.	N.D.
				Path A
		956.5	939.5 923.5	[M + NH_4_ − NH_3_]^+^ [M + NH_4_ − NH_3_ − 16 Da]^+^
				Path B
			938.5	[M + NH_4_ − H_2_O]^+^
				Path C
			920.5	[M + NH_4_ − 2H_2_O]^+^
			902.5	[M + NH_4_ − 3H_2_O]^+^

Ammoniated adducts [M + NH_4_] were subjected to fragmentation analysis

Taken together, both short-chain and long-chain polyisoprenoid alcohols were prone to oxidation with singlet oxygen upon ‘light conditions’ with formation of complex mixture of products such as hydroxides, peroxides, oxetanes and/or other cyclized derivatives.

### Oxidation of Prenol-10 with Oxygen Generated from Hydrogen Peroxide

Various methods were tested to achieve oxidation of Pren-10 with hydrogen peroxide, and finally, application of concentrated (50 %) H_2_O_2_ in a 2-propanol and ethanol mixture led to formation of oxidized derivatives of P-10 (Supplemental Figure 3). H_2_O_2_, as a thermodynamically unstable molecule, is decomposed to water and oxygen [34]. Furthermore, the rate of H_2_O_2_ decomposition increases with rising temperature. Thus, in this experiment, oxygen was an oxidizing factor formed during degradation of hydrogen peroxide.

The influence of concentrated hydrogen peroxide (50 %) on P-10 indicated almost complete degradation of the substrate (approx. 10 % left after 24 h), which was further increased when the temperature was raised (Supplemental Figure 3). MS analysis of products is summarized in Supplemental Table 4. The obtained data suggest that numerous oxidized products were formed during P-10 treatment with hydrogen peroxide (50 %) upon heating. Although identification of the oxidized P-10 products formed in these conditions requires further study, hydroxy rather than epoxy structures might be postulated based on MS/MS analysis.

### Oxidation of Prenyl Alcohols with Hydroxyl Radicals Generated Upon AOPs

In order to explore further the oxidizing potential of hydrogen peroxide on isoprenoid alcohols, various ^·^OH-generating systems, advanced oxidation processes (AOPs) [35], were used. Thu,s the combined effect of H_2_O_2_/sonication (H_2_O_2_/US) or UV/H_2_O_2_ or UV/TiO_2_ was followed (Supplemental Figure 4, Supplemental Table 5).

Those observations clearly indicate that prenyl alcohols are vulnerable to oxidation by hydroxyl radicals. These oxidation processes led to the formation of oxidized derivatives, most probably hydroxy and/or peroxy derivatives.

## Discussion

Previously, isoprenoid alcohols have been shown to undergo degradation upon singlet oxygen treatment. Reaction of citronellol with singlet oxygen generated either from hydrogen peroxide in the presence of molybdate (‘dark’ conditions) or in the presence of porphyrin (‘light’ conditions) yields a mixture of 7-hydroperoxy-3,7-dimethyloct-5-en-1-ol and 6-hydroperoxy-3,7-dimethyloct-7-en-1-ol [13, 36]. Upon subsequent reduction with Na_2_SO_3_, these hydroperoxides were converted to diols 3,7-dimethyloct-7-ene-1,6-diol and 3,7-dimethyloct-5-ene-1,7-diol [13].

**Fig. 3:**
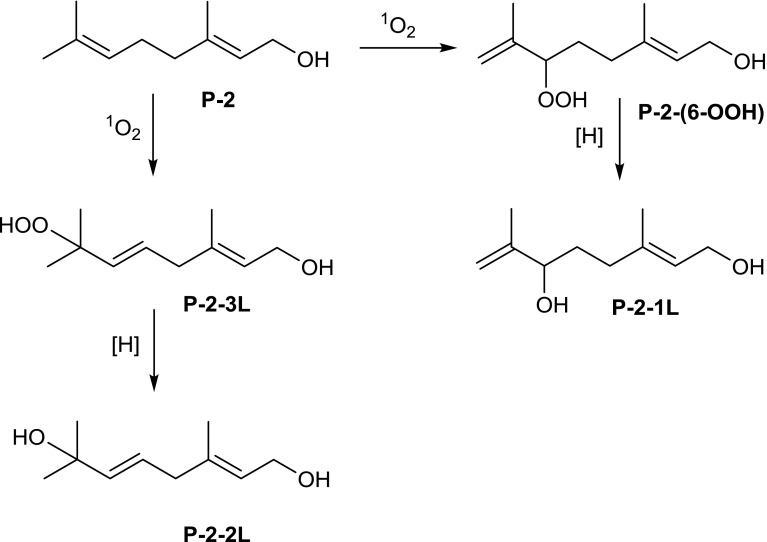
Oxidation of Prenol-2 with singlet oxygen generated in the ‘light’ conditions (in the presence of porphyrin)—proposed pathways leading to the products P-2-1L, P-2-2L, and P-2-3L

In this report, susceptibility of short- (Pren-2) and long-chain (Pren-10) prenyl alcohols towards chemical oxidation by singlet oxygen (generated in the presence of porphyrin or molybdate) was studied. The main products obtained for oxidation of Pren-2 with singlet oxygen generated in H_2_O_2_/molybdate system were as follows: 2-(hydroxymethyl)-6-isopropenyl-3-methyl-tetrahydropyran-3-ol, 1-(5-isopropenyl-2-methyl-tetrahydrofuran-2-yl)- ethane-1,2-diol and 3-[-4-hydroperoxy-4-methyl-pent-2-enyl]-3-methyl-oxiran-2-yl]-methanol (Fig. 2). Oxidation of Pren-2 led to formation of a mixture of more structurally complex end products than those obtained for citronellol. This mirrors the higher reactivity of geraniol (allylic alcohol) than α-saturated citronellol under these conditions. These products were in accordance with the ene addition products expected for reaction with singlet oxygen [37]. On the other hand, oxidation of Pren-2 with singlet oxygen generated in the presence of porphyrin resulted in formation of (2*E*)-3,7-dimethylocta-2,7-diene-1,6-diol, (2*E*,5*E*)-3,7-dimethylocta-2,5-diene-1,7-diol and (2*E*,5*E*)-7-hydroperoxy-3,7-dimethylocta-2,5-dien-1-ol (Fig. 3). Worth noting are structural differences of products of oxidation of Pren-2 with singlet oxygen observed in the ‘light’ and ‘dark’ conditions, as indicated by different *m*/*z* of the molecular ions, as well as different fragmentation paths of products (Tables 1, 2), as clearly confirmed by NMR spectra. These structural differences most probably result from the presence of hydrogen peroxide under non-photochemical conditions—a reagent able to induce reactions distinct (e.g. epoxide formation) from these resulting from the action of singlet oxygen. Moreover, different reaction conditions (e.g. temperature, solvents), which may affect the chemical rearrangements of the reactive intermediates formed upon singlet oxygen treatment [32, 33, 38], might also contribute.

In parallel, experiments in the effects of oxygen (formed upon hydrogen peroxide dismutation) and hydroxyl radical (generated under advanced oxidation processes such as H_2_O_2_/US, UV/TiO_2_ and UV/H_2_O_2_) were conducted. Both isoprenoids were prone to oxidation and numerous oxidized derivatives were obtained.

In order to analyze complex mixtures of oxidized isoprenoid derivatives, a mass spectrometry MS/MS method was established, employing fragmentation of selected ammonium adducts and careful optimization of the applied collision energy. Structural elucidations were based on comparison of the fragmentation spectra of model compounds—aldehydes and epoxides of P-2 and P-10 with those obtained for products formed in the experimental conditions. In each case, optimal parameters of the MS/MS analysis were carefully chosen (e.g. collision energy of the ion source, type of the cationic adduct).

Ammoniated and lithiated ions of isoprenoid derivatives appeared suitable for MS/MS analysis, in contrast to sodiated adducts, which were resistant to fragmentation at the applied experimental conditions. These observations were in line with the fragmentation of the lithiated prenyl ions described earlier [26].

The structures of products of P-2 and P-10 obtained during oxidations were proposed based on the fragmentation path. The pattern of the daughter ions observed for each of the analyzed compounds provides data useful for its identification, e.g. comparison of the CID spectra of ammoniated ion of the analyzed oxidized product and model compound (prenyl epoxide) indicated differences in the fragmentation path and suggested that hydroxyl rather than epoxy structure is present in this product. Furthermore, the value of the collision energy required for such fragmentation is a specific feature useful for product identification. Thus, the necessity of application of the higher collision energy (CE 30–50 eV) for analysis of oxidized products of isoprenoid alcohols, exceeding 30 eV used for P-2, P-10 and their epoxides, argues for the formation of oxidized products different than epoxides. Thus, it may be suggested that the main identified oxidized products of P-2 and P-10 recorded upon reactions with singlet oxygen were hydroxy, peroxy and heterocyclic compounds.

Such a profile of products was similar to those described in the literature for oxidation of terpenes and olefins with singlet oxygen, since hydroperoxides and diols were obtained in the presence of molybdate [13, 39], while dioxetanes, endoperoxides or allylic hydroperoxides were obtained in the presence of porphyrin [32, 33]. On the other hand, upon reaction of terpenes, nerol and geraniol with singlet oxygen generated from hydrogen peroxide in the presence of metalloporphyrin complex in homogenous system, oxidation led to formation of a mixture of products: epoxides, aldehydes and ketones [38].

Additionally, it is worth to remember that oxidation of Pren-10 with singlet oxygen using the H_2_O_2_/molybdate method did not reveal any product. This was most probably due to the relatively high hydrophobicity of the substrate, Pren-10, and consequently its low availability to singlet oxygen, which in this case was generated in a hydrophilic milieu. According to the literature, reaction with singlet oxygen generated in the H_2_O_2_/molybdate system was effective for oxidation of citronellol to produce rose oxide via the formation of hydroperoxides and diols [13] and to obtain several other products [14]. The reaction is claimed to proceed most efficiently in methanol or ethanol with Li_2_MoO_4_ as the catalyst [29]. Application of hydrogen peroxide/molybdate for oxidation is usually carried out for reactants that are relatively hydrophilic (β-citronellol) [13], whereas progress of the reaction is very slow for hydrophobic substrates (β-pinene) [40].

Oxidation of P-2 and P-10 with hydroxyl radicals revealed peroxides and hydroxides independently on the applied hydroxyl radical generating system systems (US/H_2_O_2_, UV/H_2_O_2_ or UV/TiO_2_). Oxidation of terpenes with the involvement of hydroxyl radical revealed formation of complex mixtures of products [41].

Formation of oxidized polyisoprenoids seems a unavoidable consequence of their age-related accumulation in the cells [42]. Moreover, polyisoprenoids are postulated to act as a ROS-protecting shield upon adverse environmental conditions [8, 43] in the biological membranes. Surprisingly, literature data on oxidized derivatives of polyisoprenoids are extremely limited. Thus, enzymatically catalyzed formation of polyprenyl/dolichyl aldehyde [44] and dolichoic acid [45] has been observed in yeast and mammalian cells, respectively. Moreover, the occurrence of epoxy-dolichol was documented recently in fish liver [46], while oxidized isoprenoids were also detected in human brain [47], although the mechanism, enzymatic or non-enzymatic, responsible for their synthesis remains unknown.

The oxidizing agents used in this report, singlet oxygen and hydroxyl radical, are also present in the cells. The formation of singlet oxygen and hydroxyl radical resulting from physiological processes [48] is additionally stimulated by adverse environmental conditions [49]. Moreover, oxidation of biomolecules with singlet oxygen leads to formation of signaling molecules, e.g. oxidation of carotenoids results in formation of β-cyclocitral, the modulator of the expression of a large set of genes [50].

Thus, it seems plausible to assume that chemical processes occurring in living cells might lead to the formation of products similar to those obtained in vitro, and consequently that polyisoprenoids might act as ^1^O_2_ quenchers in concert with carotenoids. Such a statement is substantiated by the occurrence of their oxy-derivatives [46, 47]. In line with these observations, exogenously applied epoxy-polyisoprenoids inhibit cholesterol and stimulate ubiquinone biosynthesis in mammalian cells [23].

The oxidation of prenyl alcohols observed in this report upon various treatments is relevant to practical aspects of everyday life, e.g. food preservation. Ultraviolet light is broadly used in food processing, disinfection of drinking water, sterilization of air and medical materials, photochemotherapy and many other practical processes. Degradation of prenyl alcohols—common components of various eukaryotic tissues and bacterial cells—should be carefully considered, although the biological role of thus formed oxidized prenyl products requires further studies. Besides, decomposition of short-chain and long-chain isoprenoids upon thermo-oxidation has been reported very recently [51]. Moreover, vulnerability of isoprenoids to oxidation should also be kept in mind in the context of environmental chemistry. Terpenes are biogenic volatile organic compounds (VOCs). Oxidation of terpenes leads to a wide variety of both gas-phase and particle-phase products that are irritating to biological tissues. Thus, terpene oxidation chemistry is considered in the literature in the context of epidemiological, toxicological and health studies, with a focus on formation and aging of secondary organic aerosol (SOA) [for review see 52].

## Electronic supplementary material

Supplementary material 1 (PDF 5352 kb)

Supplementary material 2 (PDF 117 kb)

Supplementary material 3 (PDF 284 kb)

Supplementary material 4 (PDF 223 kb)

Supplementary material 5 (DOCX 61 kb)

Supplementary material 6 (DOCX 71 kb)

Supplementary material 7 (DOCX 60 kb)

Supplementary material 8 (DOCX 62 kb)

Supplementary material 9 (DOCX 62 kb)

## Abbreviations

AOP: Advanced oxidation process
CE: Collision energy
CID: Collision-induced dissociation
D or Dol: Dolichol
EI: Electron impact
ESI–MS: Electrospray coupled with mass spectrometry
ESI–MS/MS: Electrospray coupled with tandem mass spectrometry
GC/FID: Gas chromatography coupled with flame ionization detector
GC/MS: Gas chromatography coupled with mass spectrometry
4-HNE: 4-Hydroxynonenal
HPLC/ESI–MS: High-performance liquid chromatography coupled with electrospray ionization–mass spectrometry
HPLC/UV: High-performance liquid chromatography coupled with ultraviolet absorption detector
LC/ESI–MS: Liquid chromatography coupled with electrospray ionization-mass spectrometry
MDA: Malondialdehyde
NMR: Nuclear magnetic resonance spectroscopy
P or Pren: Prenol
P-2: Prenol-2
P-10: Prenol-10
ROS: Reactive oxygen species
TLC: Thin-layer chromatography
UV: Ultraviolet radiation
