# Supplementary material for: Isoprenoid Alcohols are Susceptible to Oxidation with Singlet Oxygen and Hydroxyl Radicals
Source: Lipids. 2015 Dec 30;51:229–44. doi: 10.1007/s11745-015-4104-y (PMC4735226; doi:10.1007/s11745-015-4104-y)
Supplement: Supplementary file 1 — Supplementary material 1 (PDF 5352 kb) [file 11745_2015_4104_MOESM1_ESM.pdf]

Supplemental Figure 1.

NMR spectra of Prenol-2 oxidized products, P-2-1, P-2-2, and P-2-3 (‘dark’ conditions) and P-2-1L, P-2-2L, and P-2-3L (‘light’ conditions).

Meaning of the labels:

GER-A = P-2-1
GER-B = P-2-2
GER-D = P-2-3

Probka_A = P-2-3L
Probka_C = P-2-1L
Probka_D = P-2-2L

SpinWorks 4: GER-A CDCl3 1H

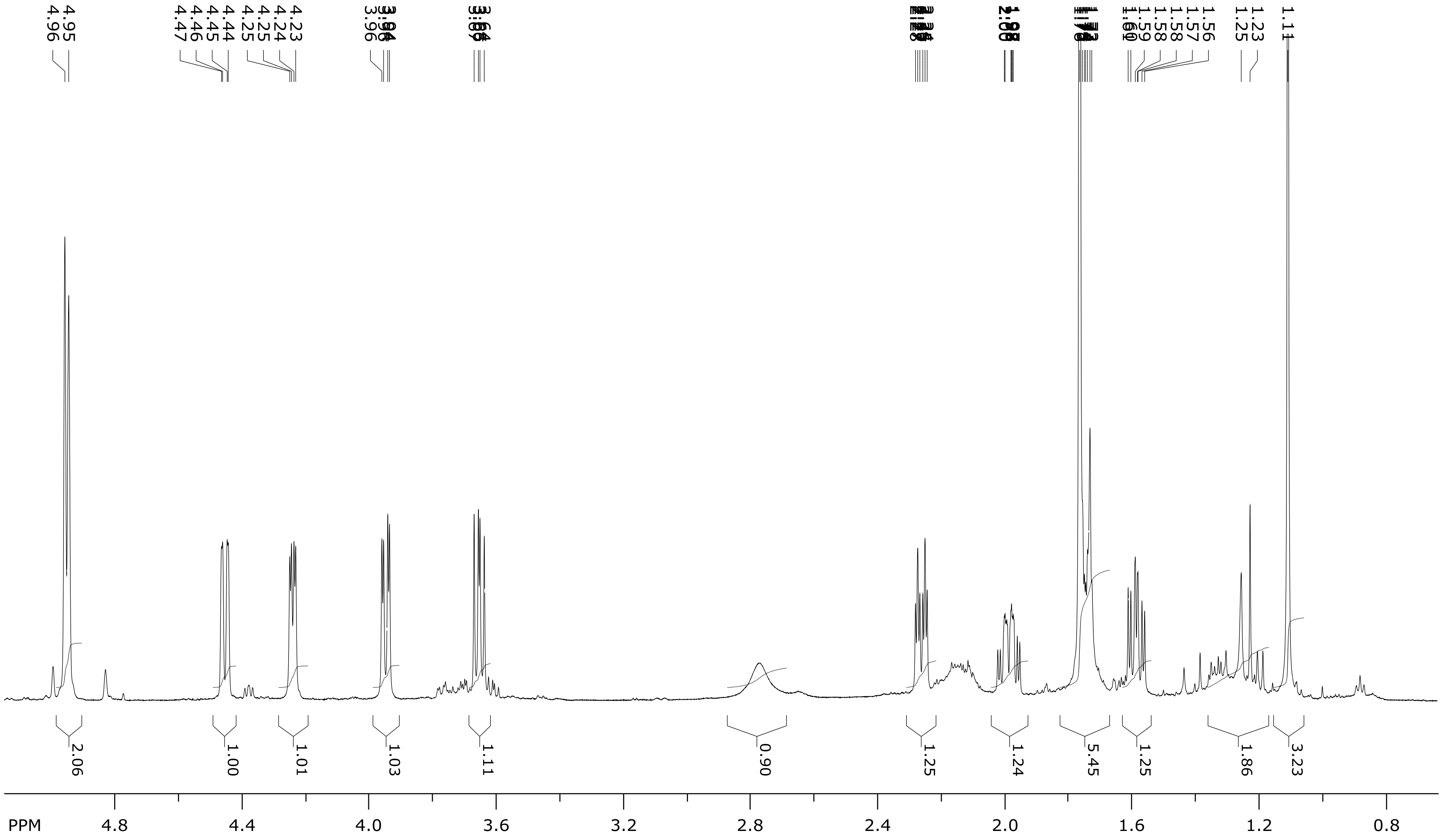

SpinWorks 4: GER-A CDCl3 13C

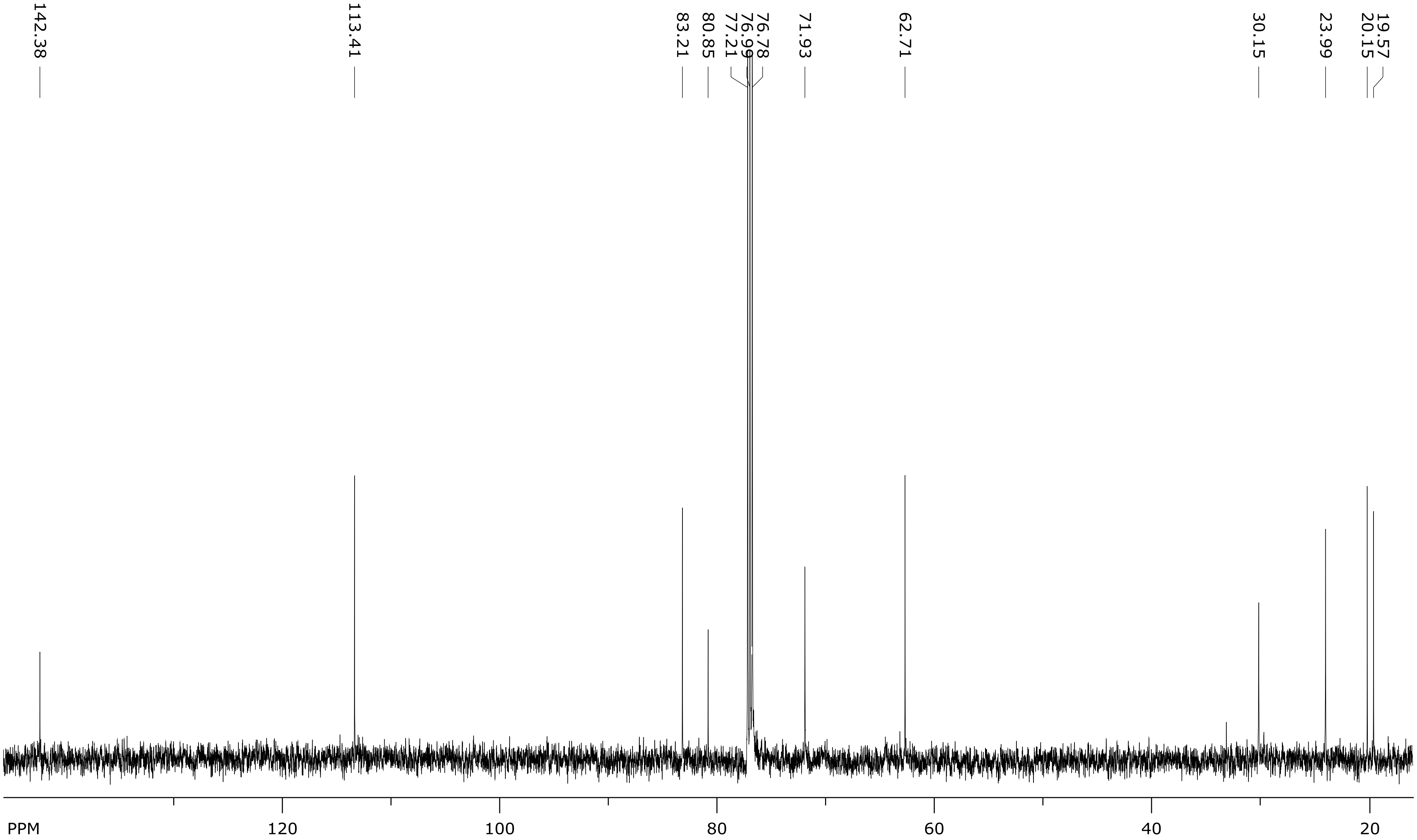

SpinWorks 4: GER-A CDCl3 1H-1H COSY

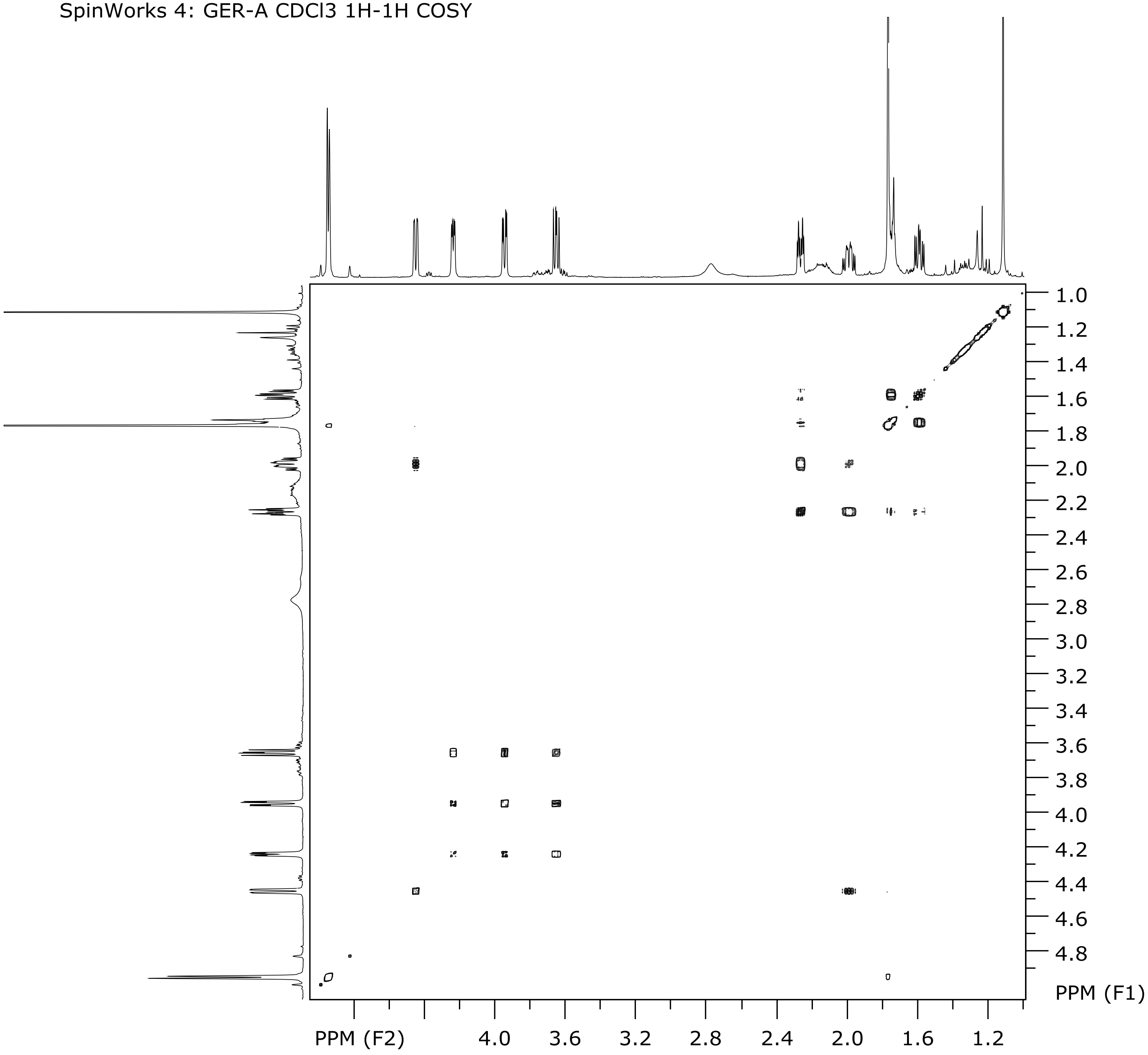

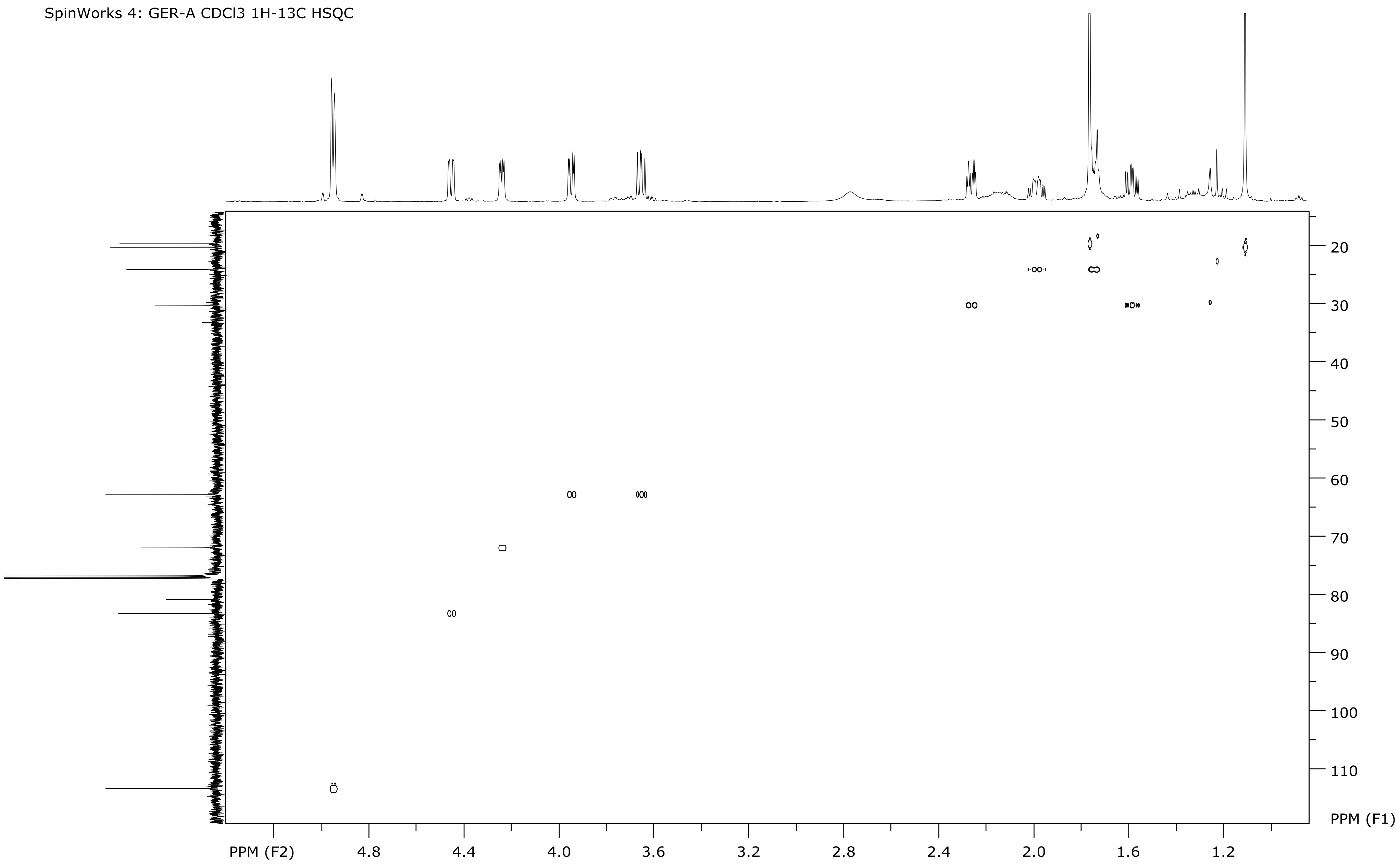
SpinWorks 4: GER-A CDCl3 1H-13C HSQC
PPM (F2)
4.8
4.4
4.0
3.6
3.2
2.8
2.4
2.0
1.6
1.2
PPM (F1)
20
30
40
50
60
70
80
90
100
110

SpinWorks 4: GER-A CDCl3 1H-13C HMBC

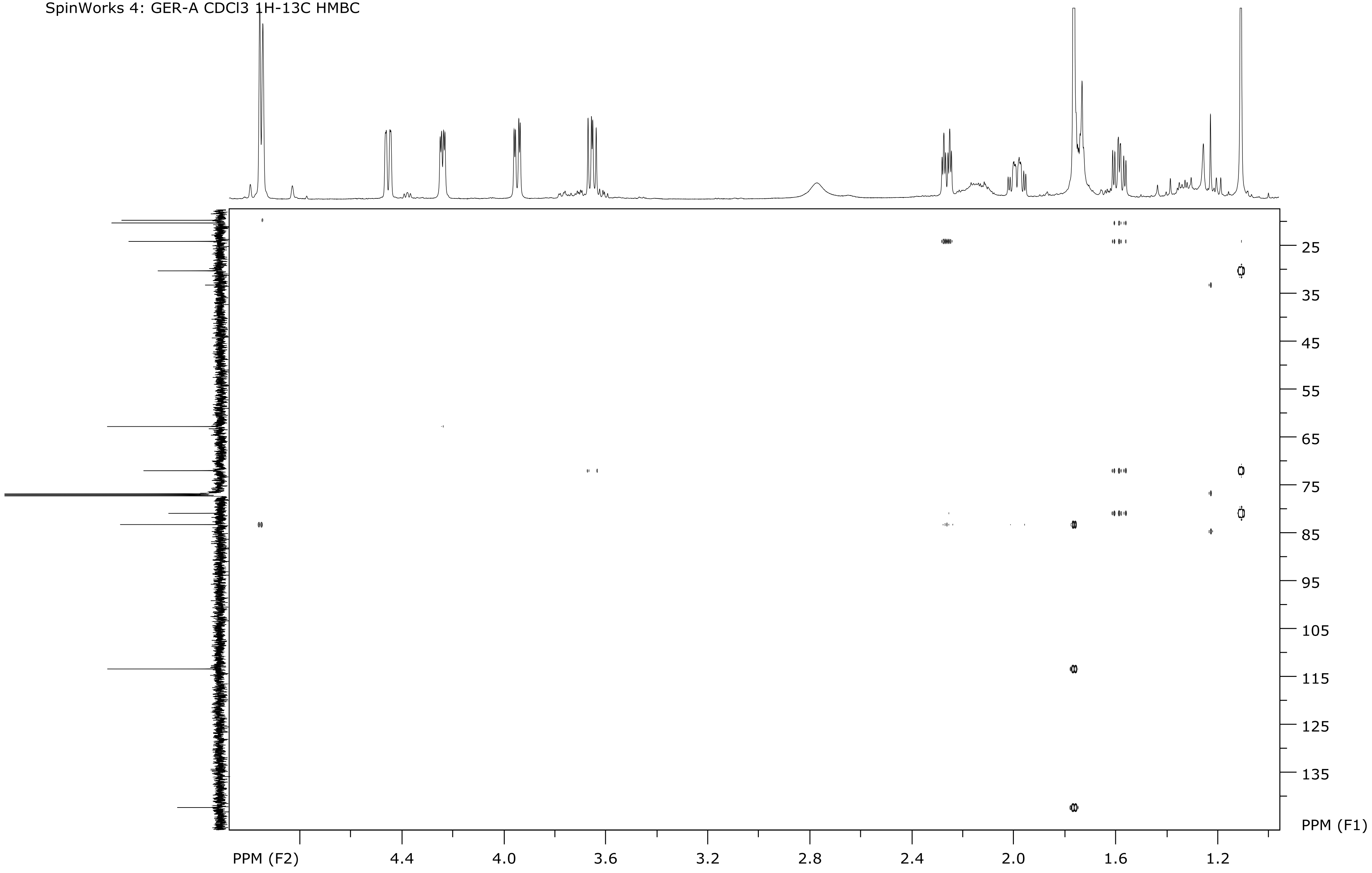

SpinWorks 4: GER-B CDCl3 1H

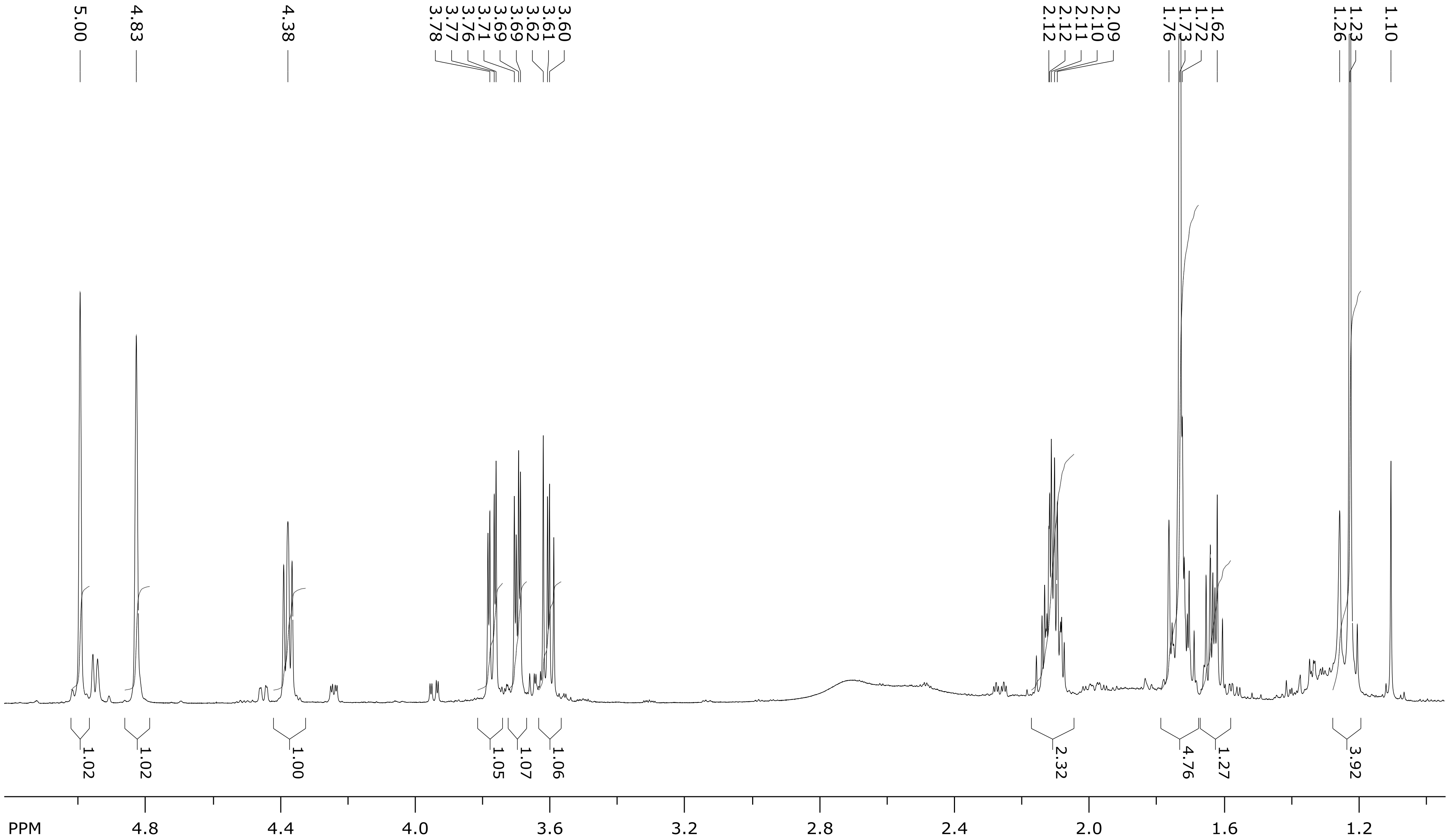

SpinWorks 4: GER-B CDCl3 13C

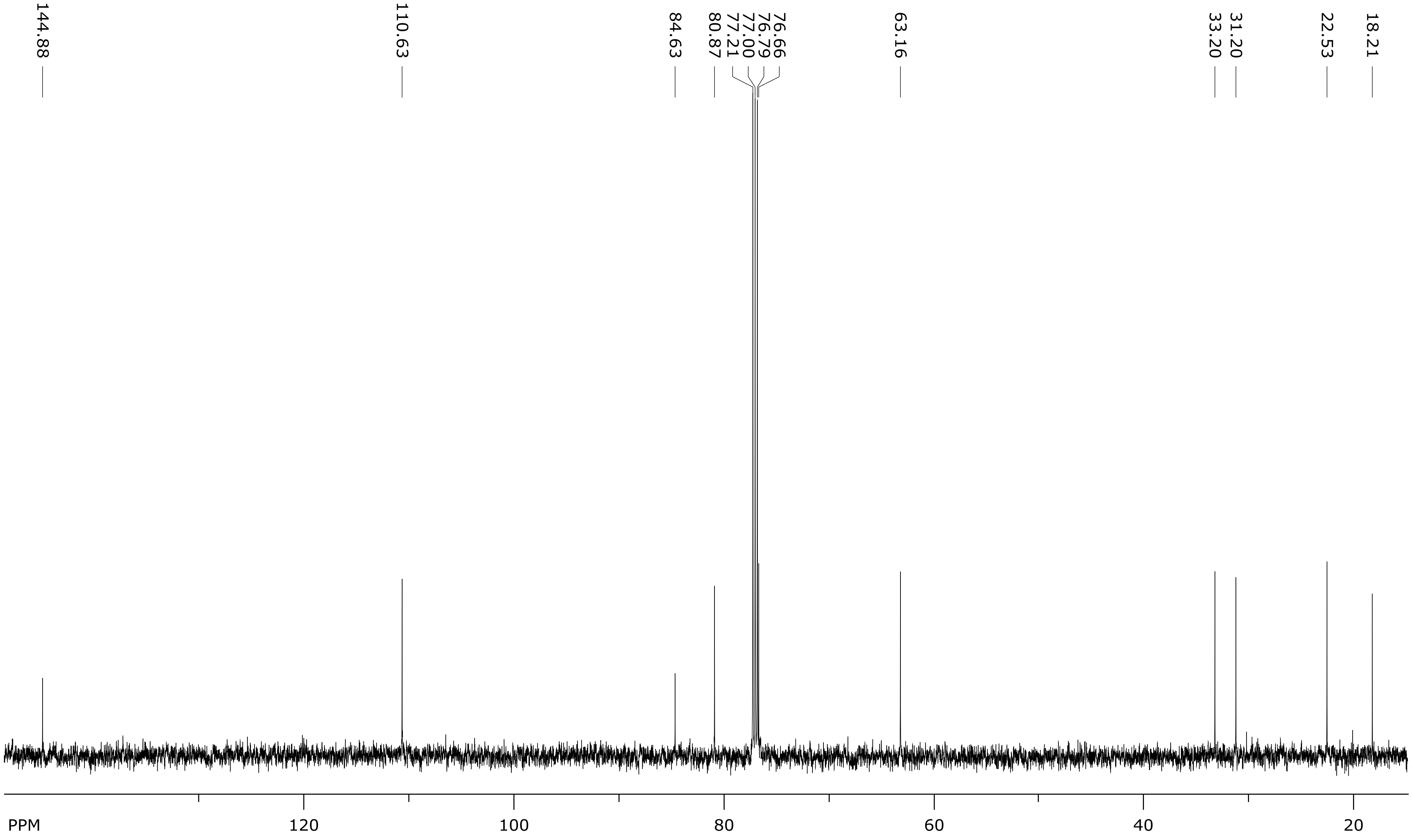

SpinWorks 4: GER-B CDCl3 1H-1H COSY

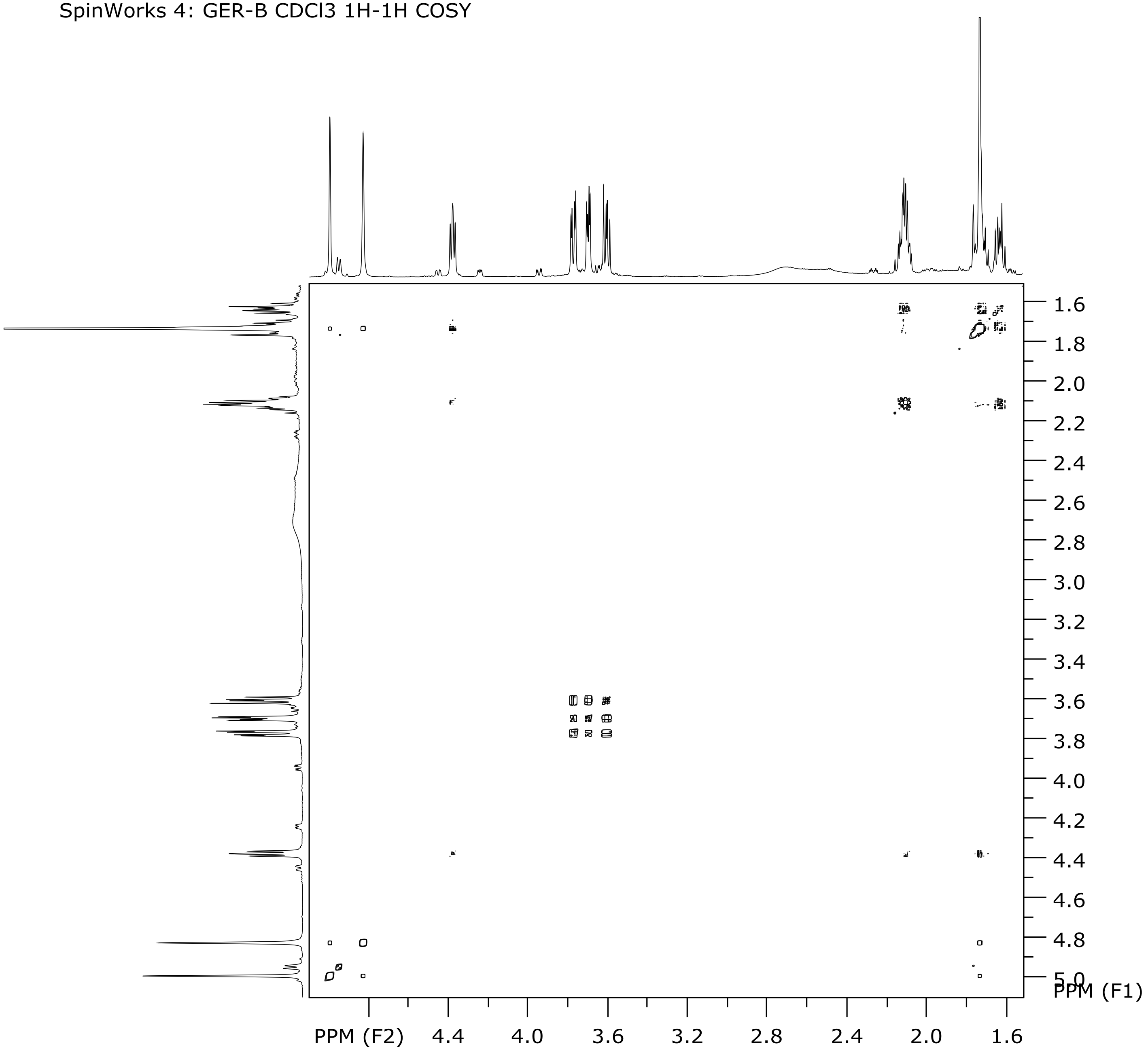

SpinWorks 4: GER-B CDCl3 1H-13C HSQC

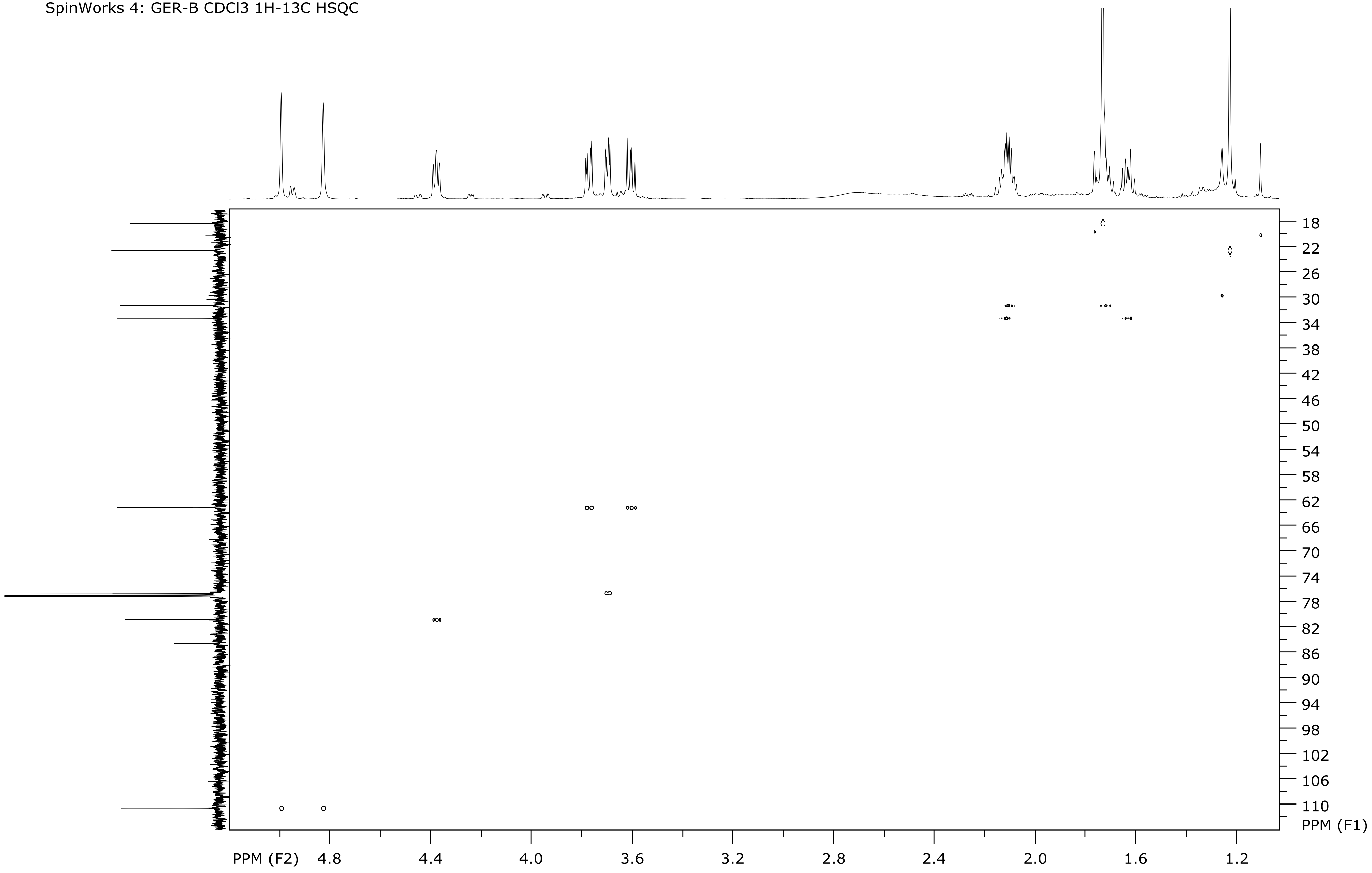

SpinWorks 4: GER-B CDCl3 HMBC

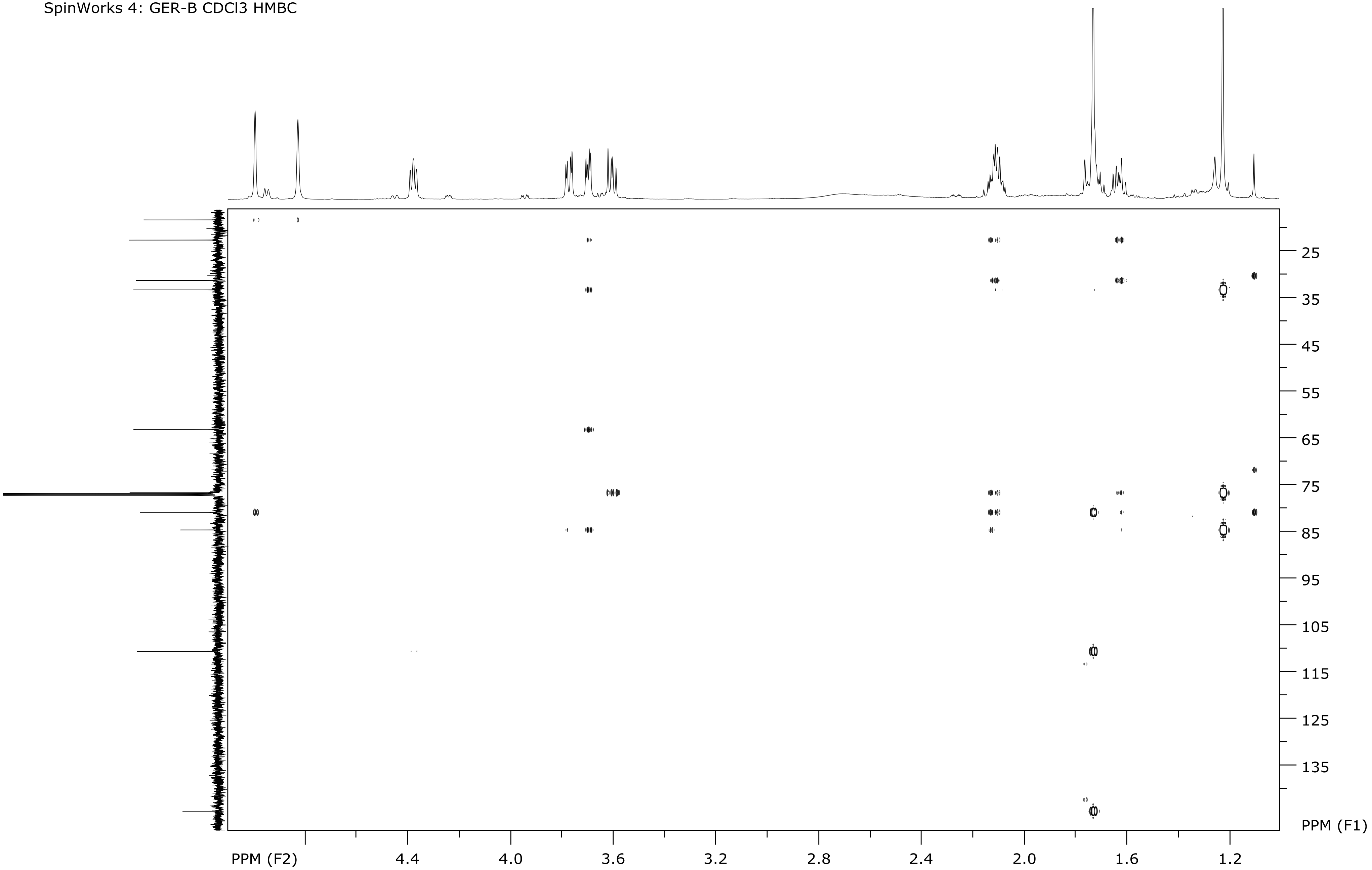

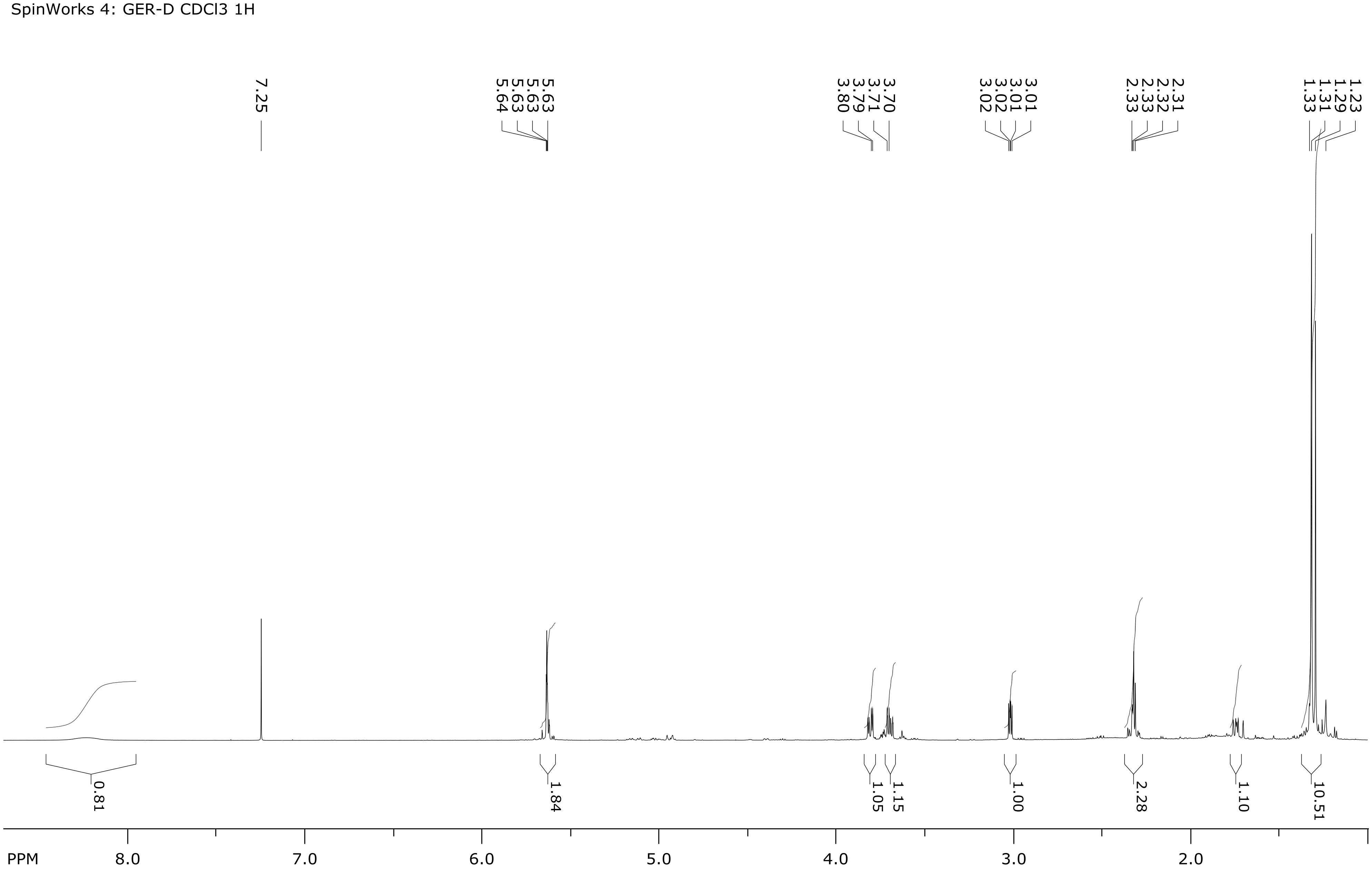
SpinWorks 4: GER-D CDCl3 1H
7.25
5.64 5.63 5.63 5.63
3.80 3.79 3.71 3.70
3.02 3.02 3.01 3.01
2.33 2.33 2.32 2.31
1.33 1.31 1.29 1.23
0.81
1.84
1.05
1.15
1.00
2.28
1.10
10.51
PPM
8.0
7.0
6.0
5.0
4.0
3.0
2.0

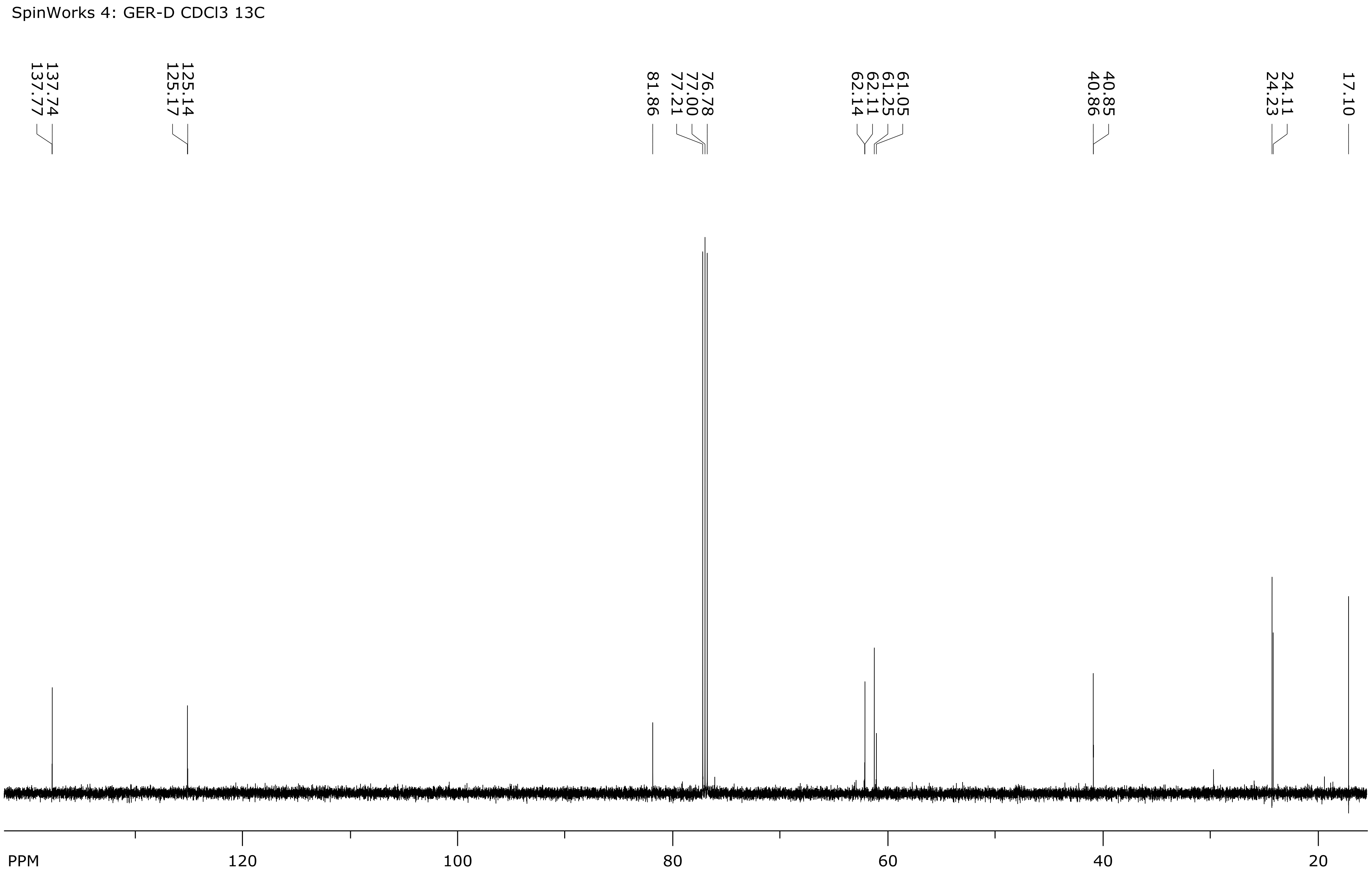
SpinWorks 4: GER-D CDCl3 13C
137.74
137.77
125.14
125.17
81.86
77.21
77.00
76.78
62.14
62.11
61.25
61.05
40.85
40.86
24.11
24.23
17.10
PPM
120
100
80
60
40
20

SpinWorks 4: GER-D CDCl3 1H-1H COSY

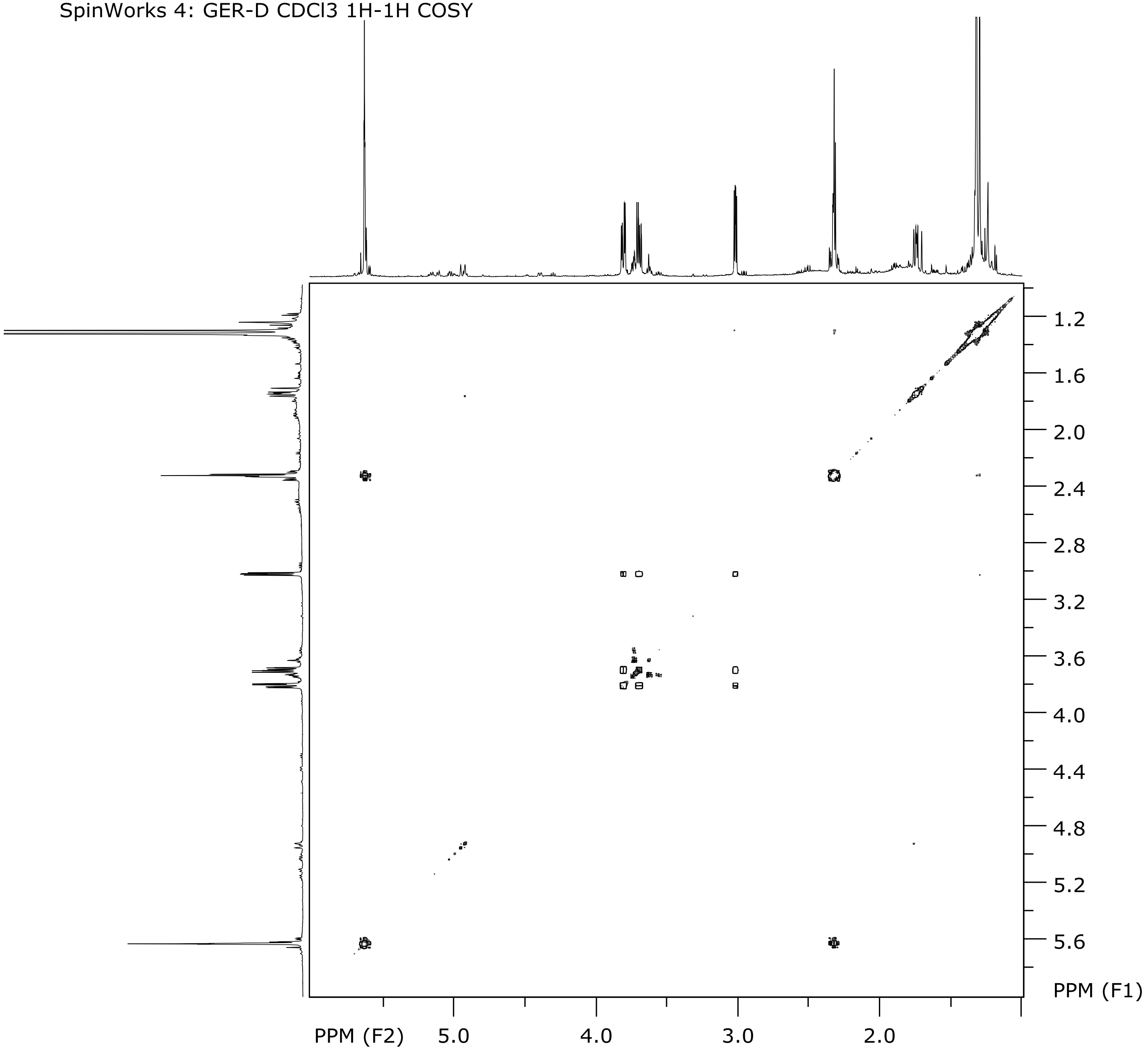

SpinWorks 4: GER-D CDCl3 HSQC

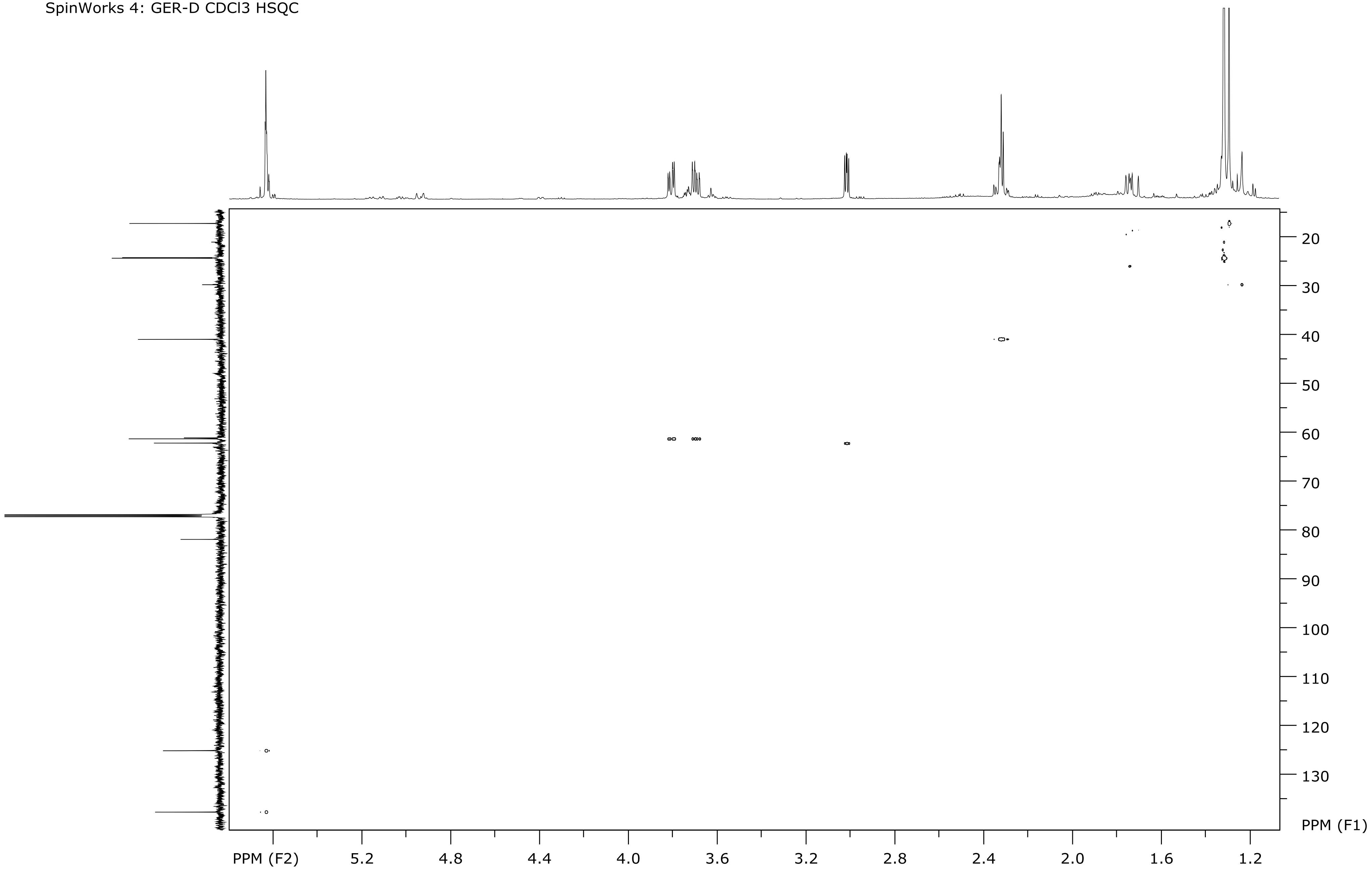

SpinWorks 4: GER-D CDCl3 HMBC

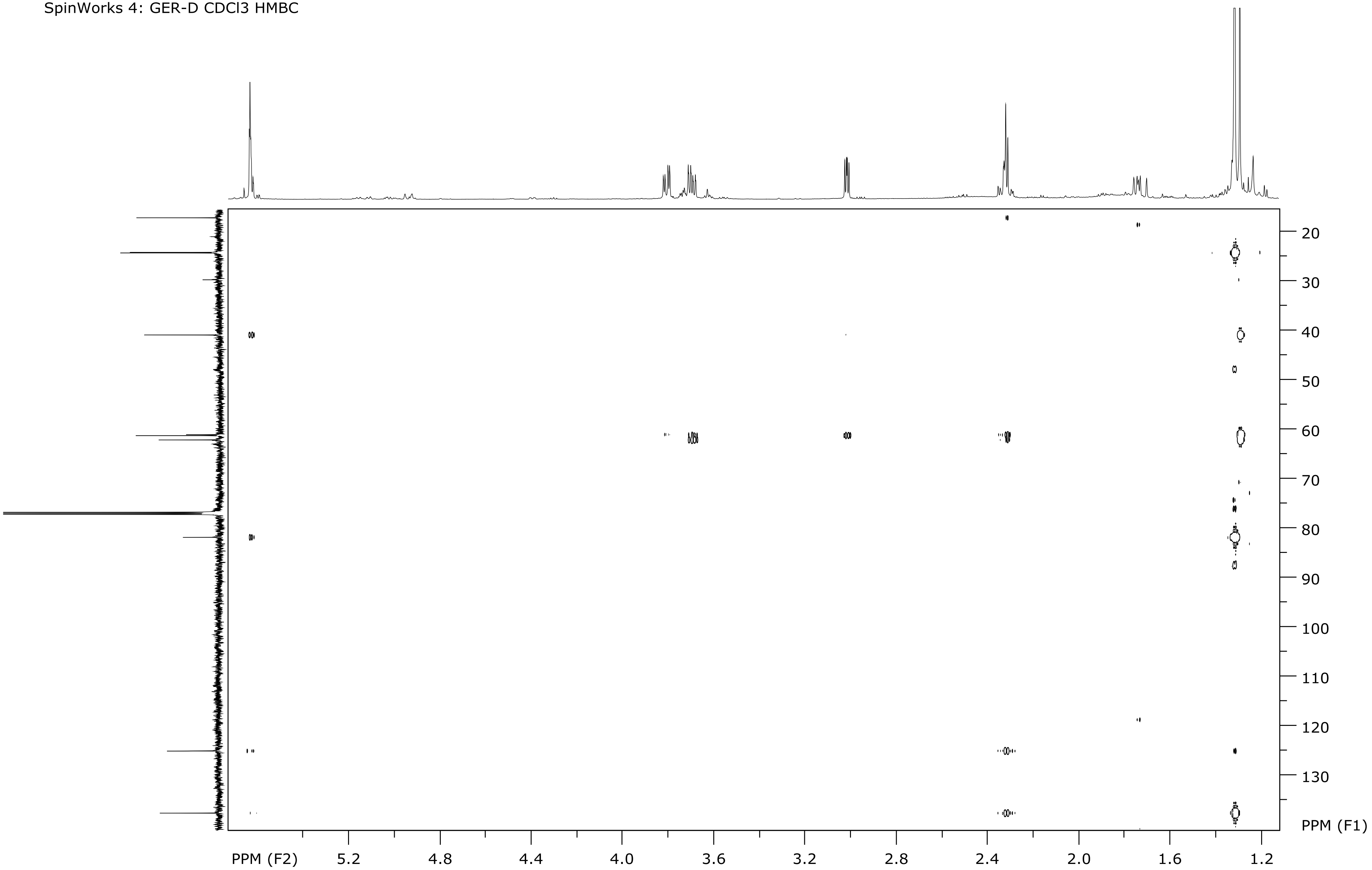

SpinWorks 4: probka_A-H1

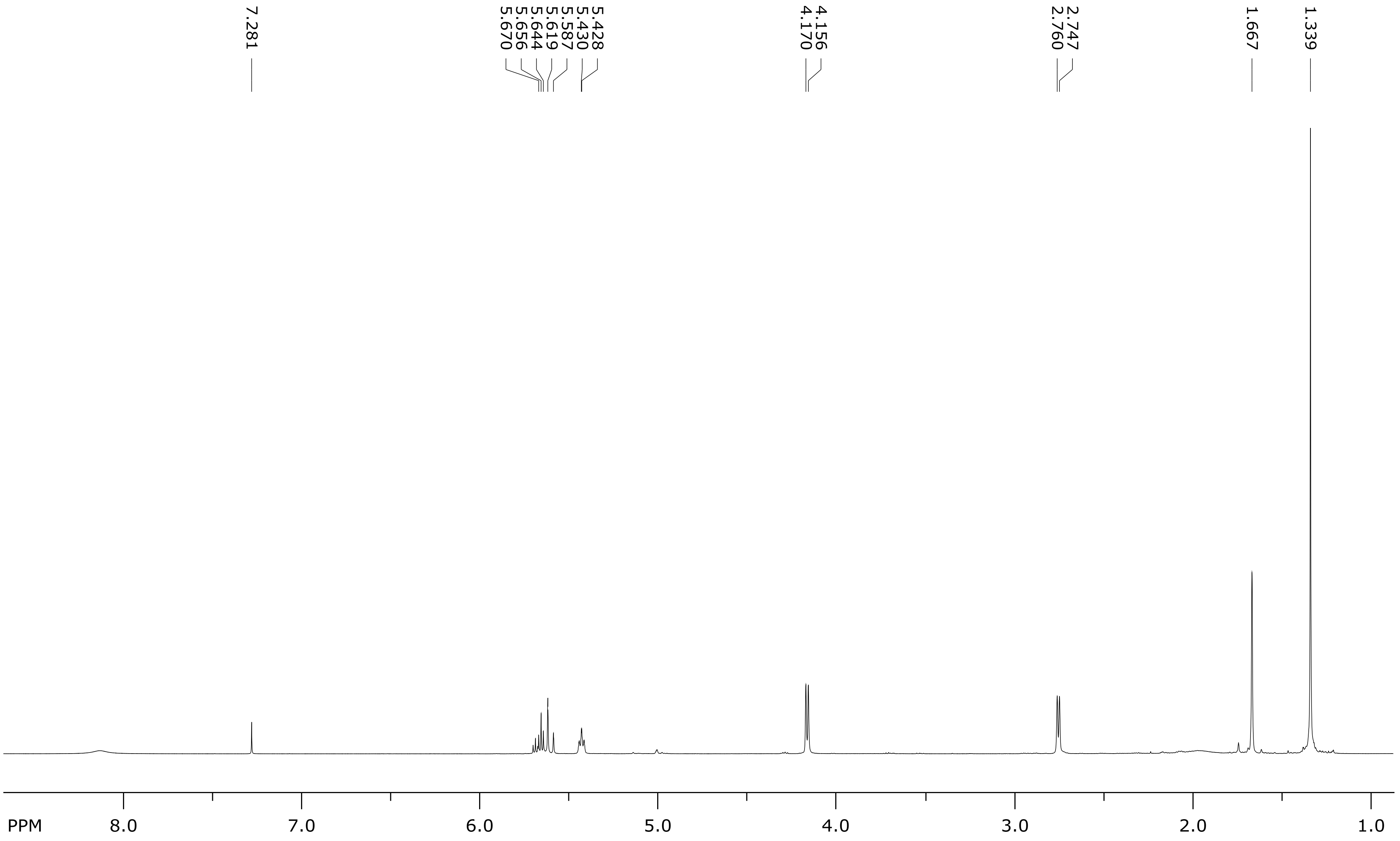

SpinWorks 4: probka_A-C13

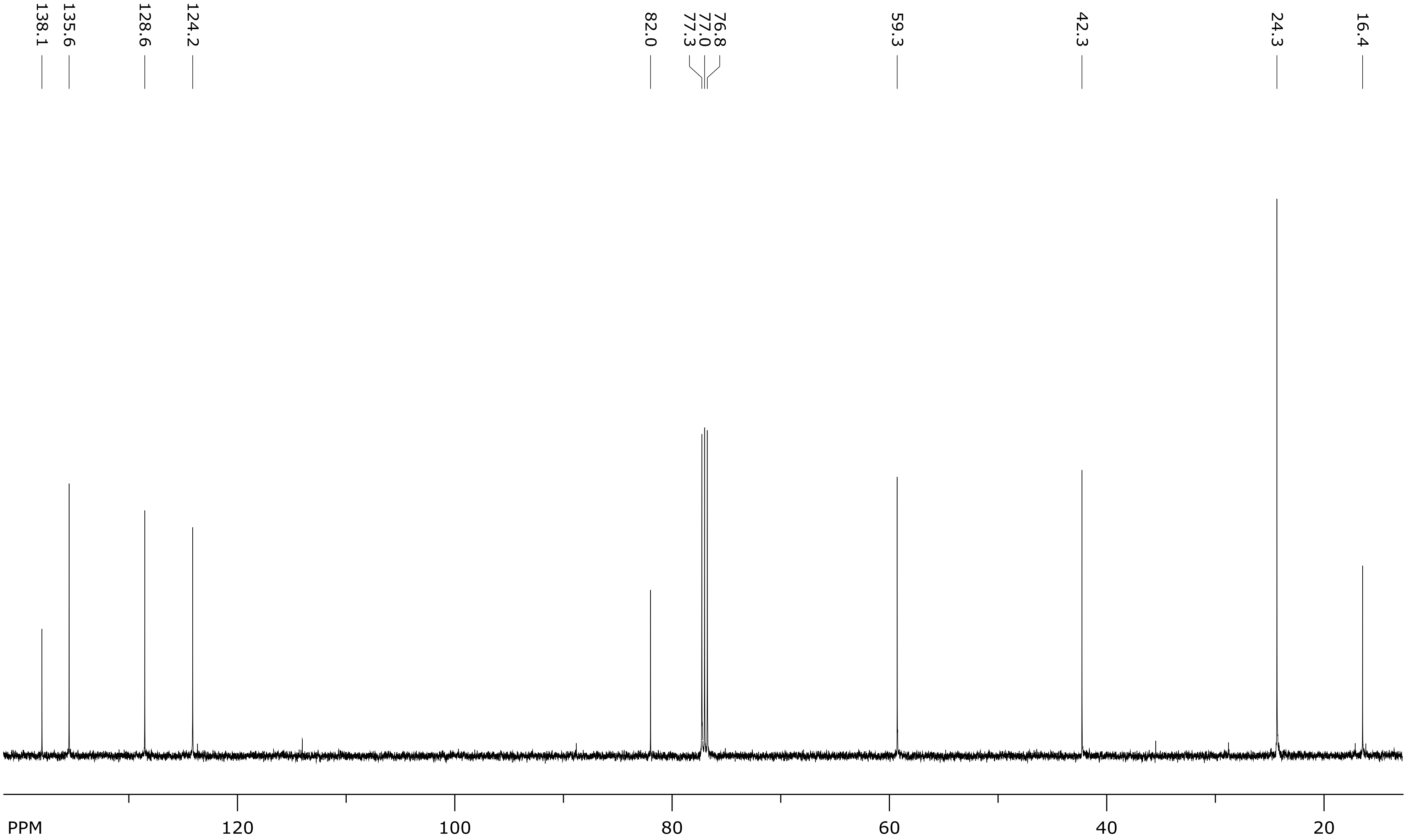

SpinWorks 4: Próbka C - 1H

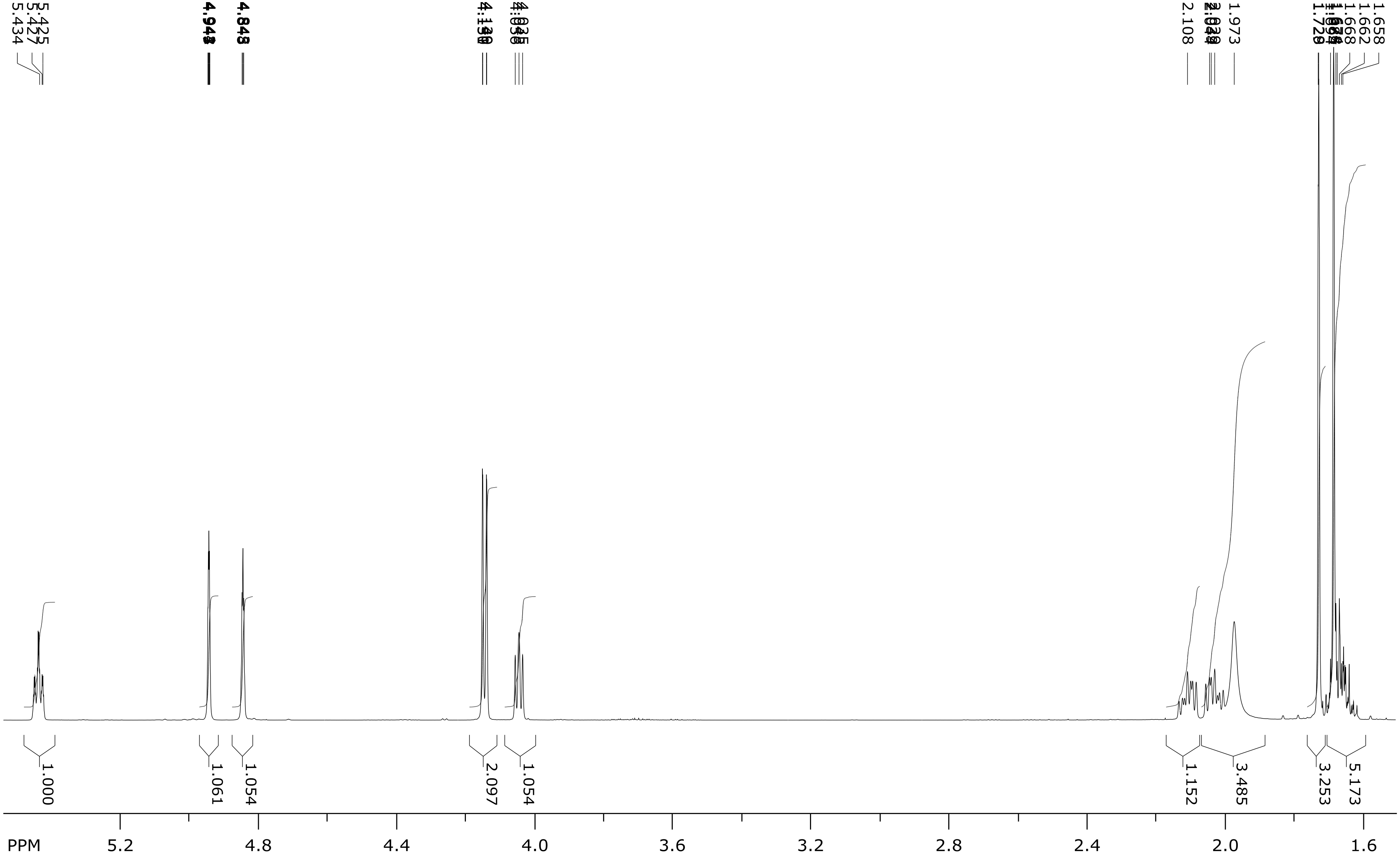

SpinWorks 4: probka C - C13

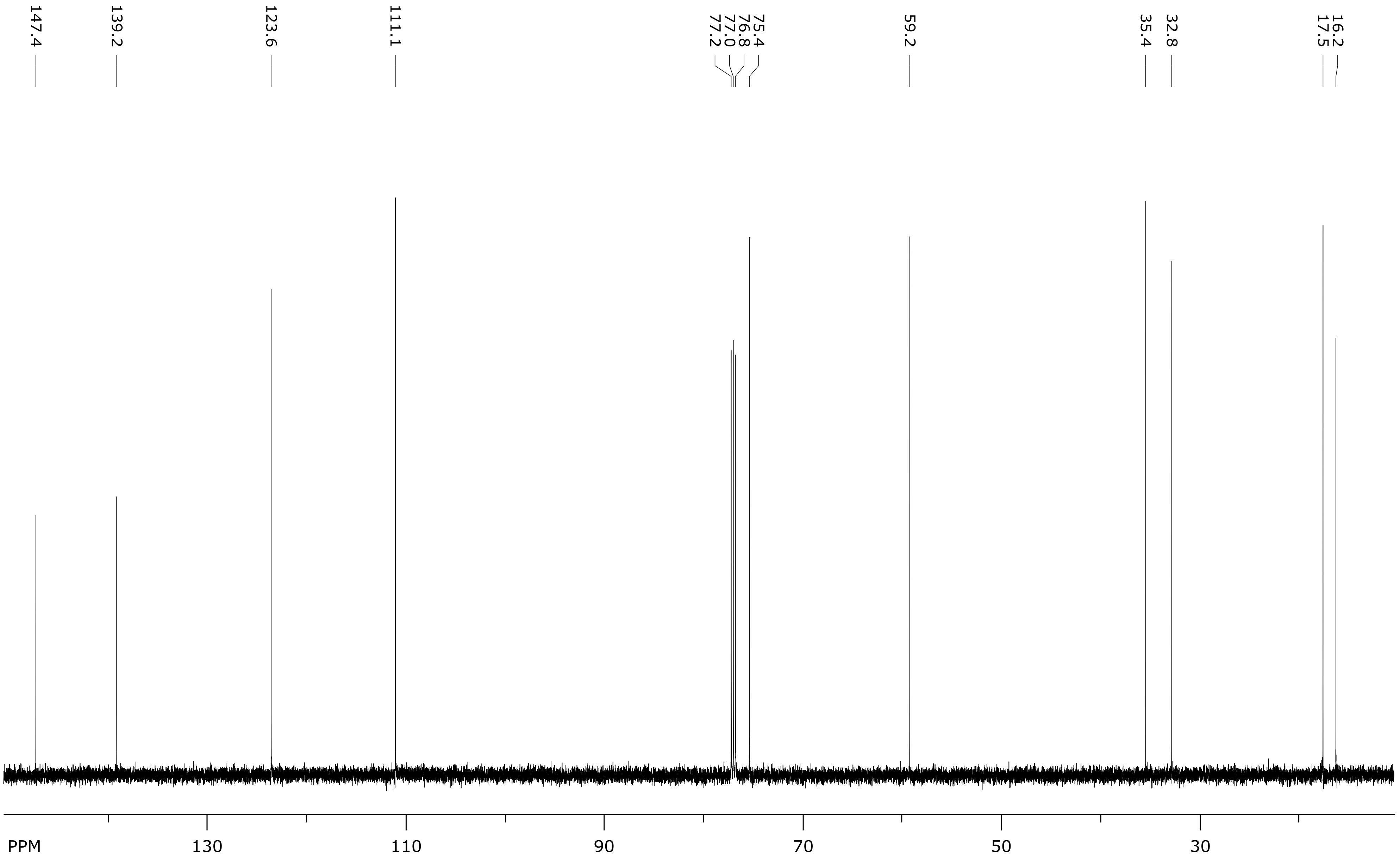

SpinWorks 4: probka_D-H1

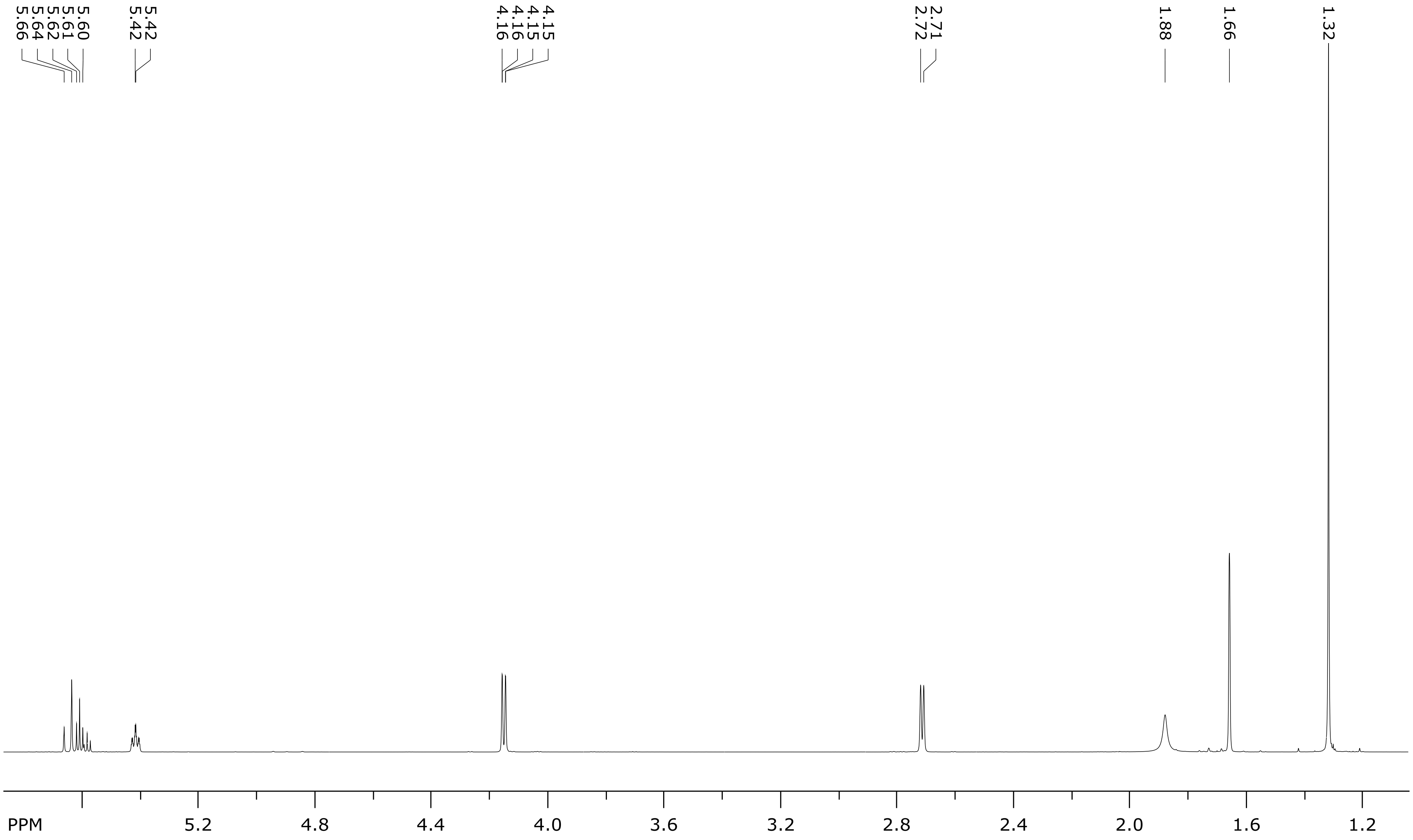

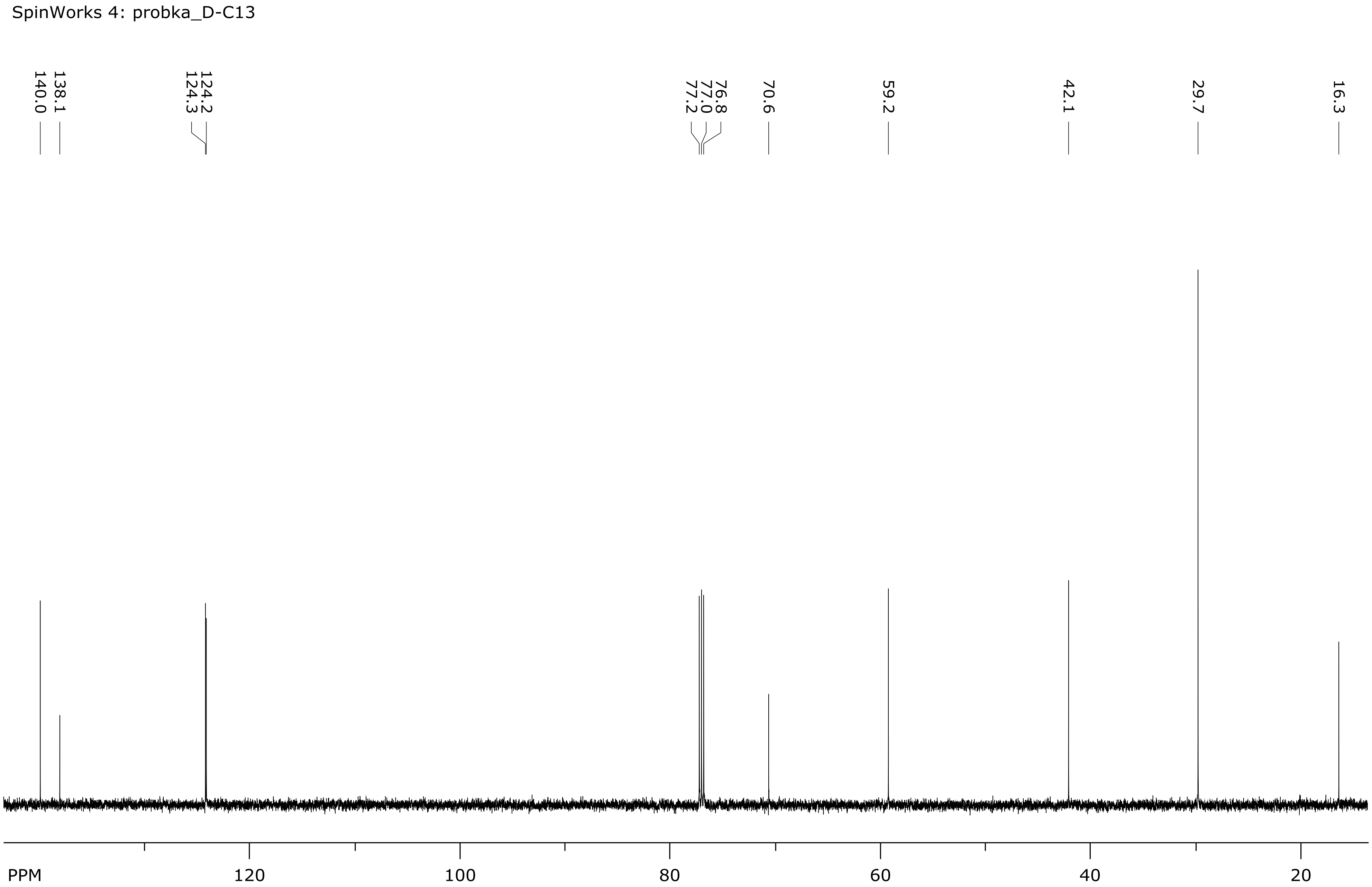
SpinWorks 4: probka_D-C13
140.0
138.1
124.3
124.2
77.2
77.0
76.8
70.6
59.2
42.1
29.7
16.3
PPM
120
100
80
60
40
20
