# Supplementary material for: Isoprenoid Alcohols are Susceptible to Oxidation with Singlet Oxygen and Hydroxyl Radicals
Source: Lipids. 2015 Dec 30;51:229–44. doi: 10.1007/s11745-015-4104-y (PMC4735226; doi:10.1007/s11745-015-4104-y)
Supplement: Supplementary file 2 — Supplementary material 2 (PDF 117 kb) [file 11745_2015_4104_MOESM2_ESM.pdf]

Supplemental Figure 2.

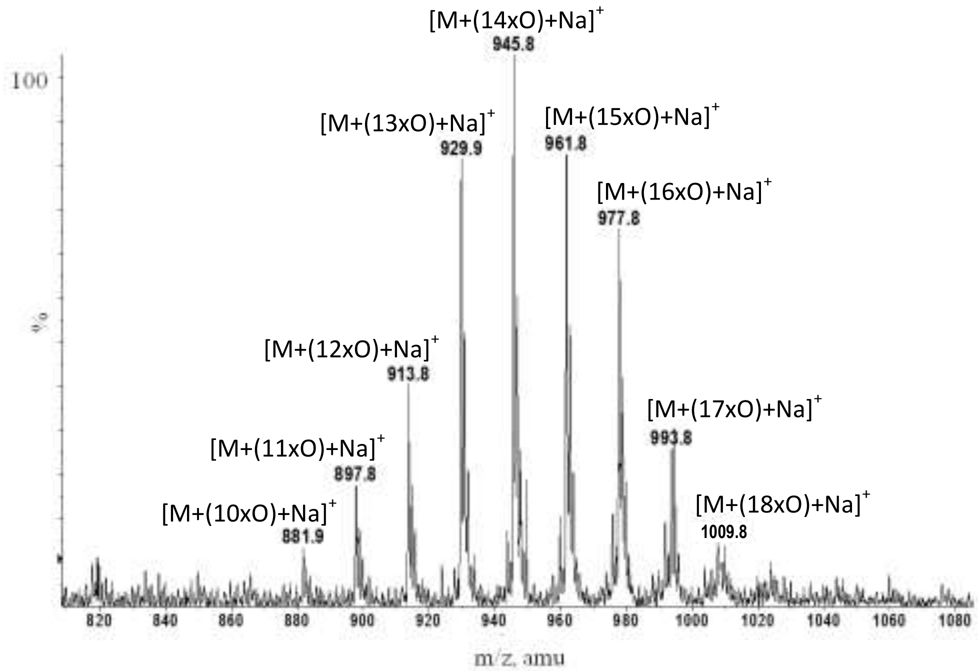

Supplemental Figure 2.

ESI-MS analysis of sodiated Pren-10 oxidation products formed upon singlet oxygen treatment (generated in the presence of porphyrin).
