# Supplementary material for: Isoprenoid Alcohols are Susceptible to Oxidation with Singlet Oxygen and Hydroxyl Radicals
Source: Lipids. 2015 Dec 30;51:229–44. doi: 10.1007/s11745-015-4104-y (PMC4735226; doi:10.1007/s11745-015-4104-y)
Supplement: Supplementary file 3 — Supplementary material 3 (PDF 284 kb) [file 11745_2015_4104_MOESM3_ESM.pdf]

Supplemental Figure 3

(A)

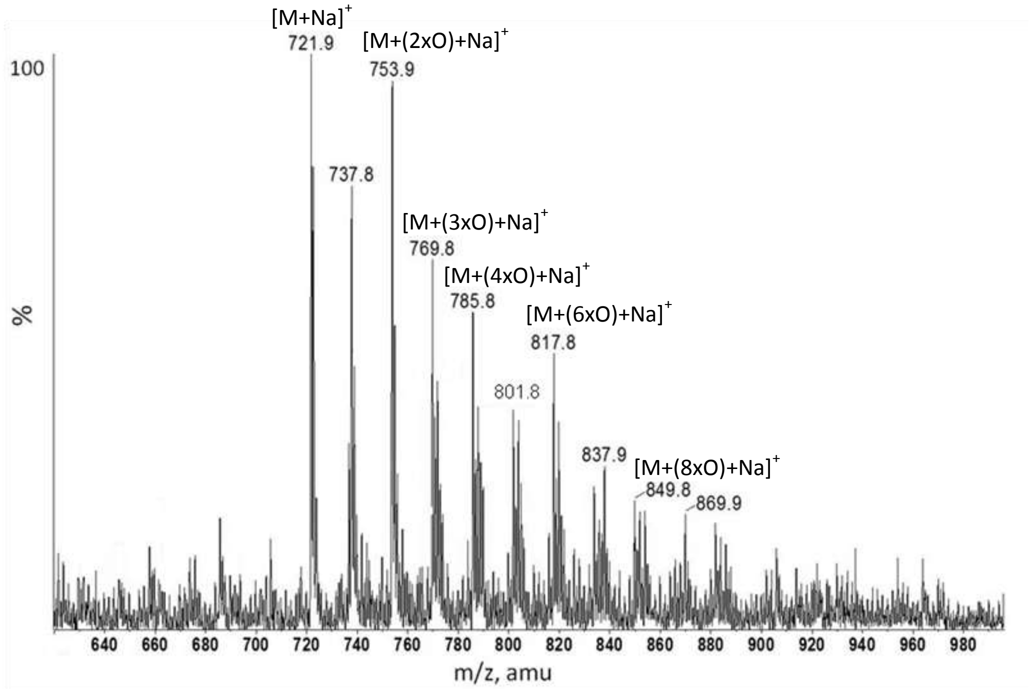

(B)

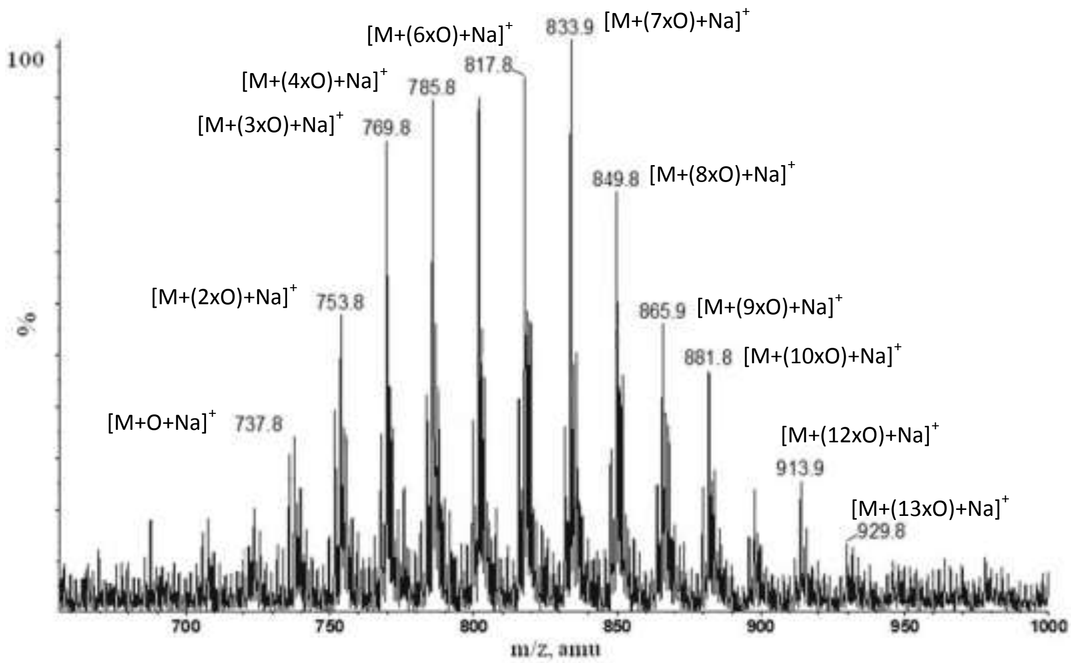

(C)

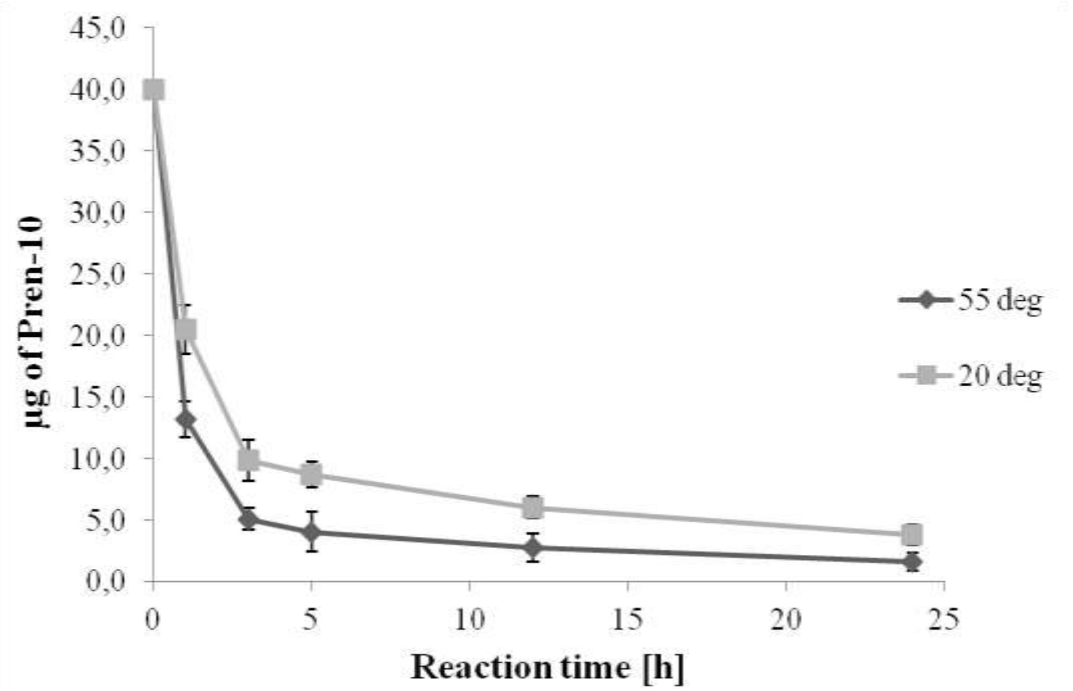

Supplemental Figure 3. Oxidation of Prenol-10 upon hydrogen peroxide treatment at 55 °C for (A) 1 h and (B) 12 h**.** Formed products were analyzed by ESI-MS, signals corresponding to oxidized Pren-10 molecules containing increasing number of oxygen atoms (sodiated ions) are indicated.

(C) Degradation rate of Prenol-10 upon hydrogen peroxide treatment - effects of lower (20 °C) (squares), and higher temperature (55 °C) (diamonds) was compared. Content of the substrate (Prenol-10) was followed by HPLC/UV.
