# Supplementary material for: Isoprenoid Alcohols are Susceptible to Oxidation with Singlet Oxygen and Hydroxyl Radicals
Source: Lipids. 2015 Dec 30;51:229–44. doi: 10.1007/s11745-015-4104-y (PMC4735226; doi:10.1007/s11745-015-4104-y)
Supplement: Supplementary file 4 — Supplementary material 4 (PDF 223 kb) [file 11745_2015_4104_MOESM4_ESM.pdf]

Supplemental Figure 4.

(A)

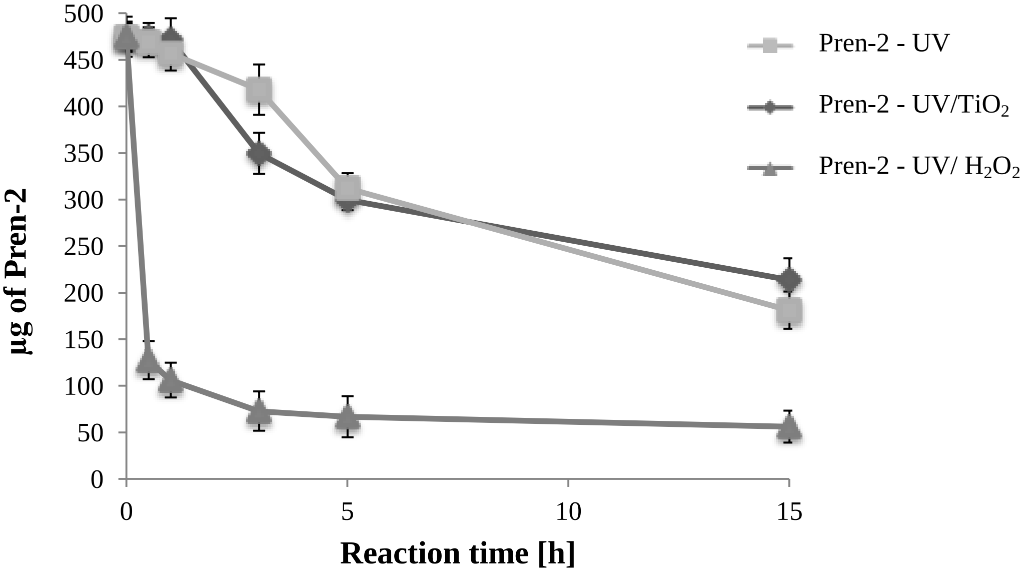

(B)

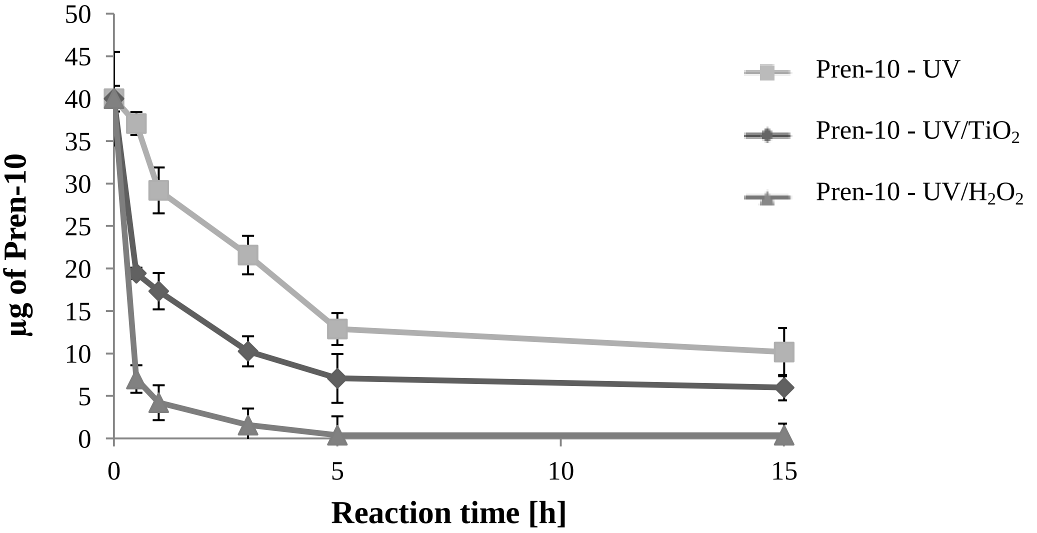

(C)

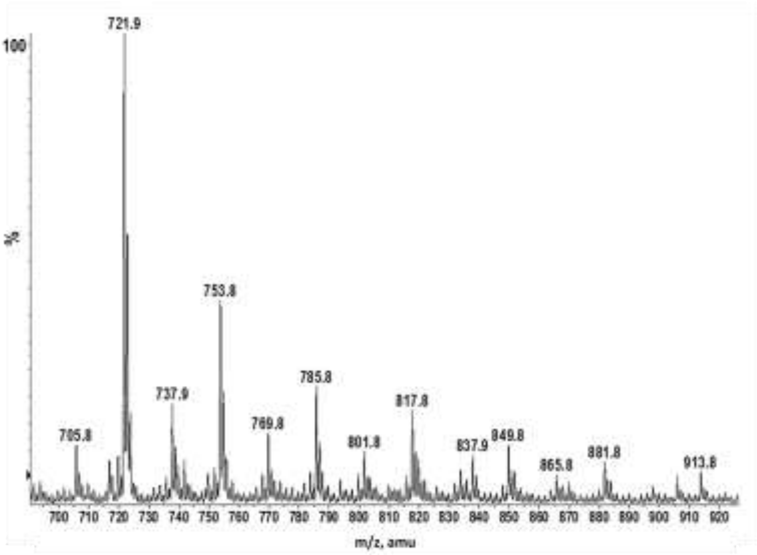

(D)

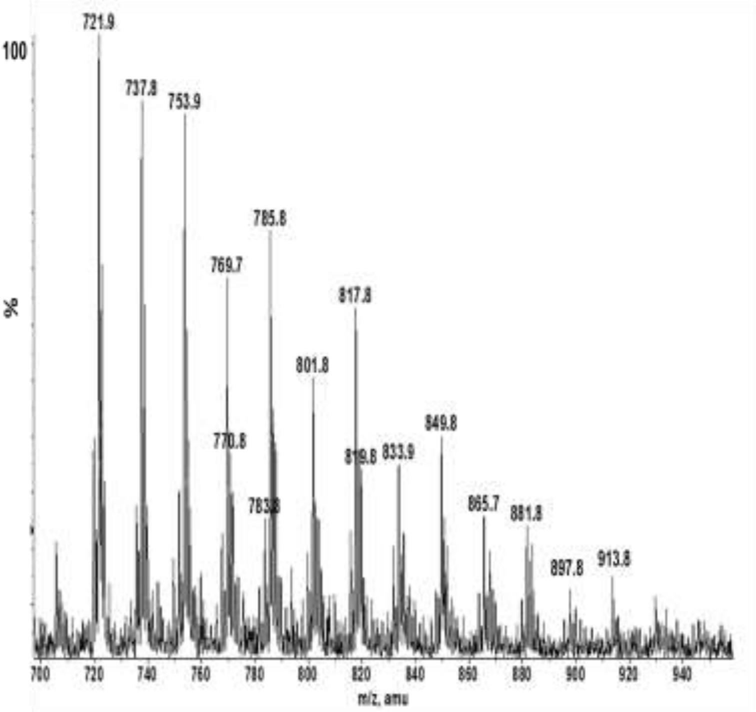

Supplemental Figure 4. **Oxidation of prenyl alcohols with hydroxyl radicals generated upon the combined effect of UV light, hydrogen peroxide or titanium dioxide.**

Degradation rate of prenyl alcohols Prenol-2 (A) and Prenol-10 (B) upon UV treatment - effects of UV alone (squares), combined UV / titanium oxide (diamonds) and UV / hydrogen

peroxide (triangles) treatments. Content of the substrate Prenol-2 and Prenol-10 was followed by GC/FID and HPLC/UV, respectively.
Oxidation of Prenol-10 upon (C) sole UV and (D) combined UV / hydrogen peroxide treatments. Products formed after 5 h of irradiation were analyzed by ESI-MS, signals corresponding to oxidized Pren-10 molecules containing increasing number of oxygen atoms (sodiated ions) are indicated.

Comments to Supplemental Figure 4.
UV treatment was performed as a control and led to degradation of P-10, which was considerably faster than P-2 since 3h and 9h were required for degradation of half of the initial amount of P-10 and P-2, respectively (Supplemental Figure 4A, B). Based on the degradation rate, it was confirmed that both P-10 and P-2, were susceptible to UV light treatment.
Treatment of P-2 and P-10 with UV/hydrogen peroxide caused fast degradation of both substrates with simultaneous increase of the formation of oxidation products. After 3 h of irradiation in the presence of H_2O_2 only 4 % and 15.5 % of the initial amount of P-10 and P-2, respectively, remained in the reaction mixture (Supplemental Figure 4A, B). Decomposition of P-10 was completed after 15 h of the reaction in contrast to P-2 (approx. 12 % left).
ESI-MS analysis of the products of P-2 and P-10 formed upon UV and UV/H_2O_2 treatment revealed the formation of oxidized derivatives (Supplemental Figure 4C, D), see also Supplemental Table 7.

The structures of products obtained during P-10 oxidation both under UV irradiation (Supplemental Figure 4) and UV/H_2O_2 were proposed on the basis on MS/MS analysis (CE 30 ÷ 50 eV). The CID spectrum of selected lithiated ion (*m/z* 721.5) of P-10 containing one additional oxygen atom in the molecule, revealed the loss of one water molecule (*m/z* 703.5) and subsequent isoprene residues (*m/z* 635.5 - 91.0). Parallel fragmentation path showed loss of a 12 Da molecule (*m/z* 691.5) and then probably an ethene molecule (28 Da) resulting in *m/z* 685.5.

The fragmentation spectra of the second product of P-10 (*m/z* 737.5, corresponding to $\text{Product}_{\text{P-10}}$ No 12) indicated the loss of two water molecules, respectively, (*m/z* 719.4, 701.6), which possibly suggests the presence of two additional oxygen atoms in the product molecule.

the CID spectrum of the second product of P-10 (*m/z* 753.9) revealed the loss of two subsequent water molecules and 16 Da fragment (*m/z* 735.9, 717.9, 701.8).

Observed fragmentation patterns were different than those noted for model P-10 mono- and diepoxides (Supplemental Table 2) or also for P-10 oxidized products obtained upon singlet oxygen treatment (in the presence of light and porphyrin). Moreover, collision energy required for fragmentation of these products (CE 50 eV) was significantly higher than that required for P-10 epoxides (30 eV) which again suggested that their structure was different than epoxides.
