# Supplementary material for: Isoprenoid Alcohols are Susceptible to Oxidation with Singlet Oxygen and Hydroxyl Radicals
Source: Lipids. 2015 Dec 30;51:229–44. doi: 10.1007/s11745-015-4104-y (PMC4735226; doi:10.1007/s11745-015-4104-y)
Supplement: Supplementary file 5 — Supplementary material 5 (DOCX 61 kb) [file 11745_2015_4104_MOESM5_ESM.docx]

Supplemental Table 1. Pren-2 and its oxidized standards (Prenal-2, monoepoxy, diepoxy-prenol-2) - mass spectrometry analysis (ESI-MS and ESI-MS/MS). Ammoniated adducts [M + NH_4_] were subjected to fragmentation analysis. MW 154.0 for Prenol-2.

| Products of Pren-2 | Molecular ion  *m/z* | |  | MS/MS analysis – daughter ions | |
| --- | --- | --- | --- | --- | --- |
|  | [M + Na]^+^ | [M + NH_4_]^+^ |  | *m/z* | fragmentation path |
| Prenol-2  (M_P-2_) | 177.0 |  |  | N.D. | N.D. |
|  |  | 172.0 |  | 155.0  137.0  109.0 | [M_P-2_ + NH_4_ - NH_3_]^+^  [M_P-2_ + NH_4_ - NH_3_ - H_2_O]^+^  [M_P-2_ + NH_4_ - NH_3_ - H_2_O - 28 Da]^+^ |
| Prenal-2  (M_P-2-al_) | 175.0 |  |  | N.D. | N.D. |
|  |  | 170.0 |  | 153.0  135.0  107.0 | [M_P-2-al_ + NH_4_ - NH_3_]  [M_P-2-al_ + NH_4_ - NH_3_ - H_2_O]^+^  [M_P-2-al_ + NH_4_ - NH_3_ - H_2_O - 28 Da]^+^ |
| Prenol-2 epoxide  (M_P-2-epo_) | 193.3 |  |  | N.D. | N.D. |
|  |  | 188.1 |  | 171.0  153.0  135.0  107.0 | [M_P-2-epo_ + NH_4_ - NH_3_ ]^+^  [M_P-2-epo_ + NH_4_ - NH_3_ - H_2_O]^+^  [M_P-2-epo_ + NH_4_ - NH_3_ - 2 H_2_O]^+^  [M_P-2-epo_ + NH_4_ - NH_3_ - 2 H_2_O - 28 Da]^+^ |
| Prenol-2 diepoxide  (M_P-2-diepo_) | 209.3 |  |  | N.D. | N.D. |
|  |  | 204.1 |  | 187.0  169.0  151.0  133.0  105.0 | [M_P-2-diepo_ + NH_4_ - NH_3_]^+^  [M_P-2-diepo_ + NH_4_ - NH_3_ - H_2_O]^+^  [M_P-2-diepo_ + NH_4_ - NH_3_ - 2 H_2_O]^+^  [M_P-2-diepo_ + NH_4_ - NH_3_ - 3 H_2_O]^+^  [M_P-2-diepo_ + NH_4_ - NH_3_ - 3 H_2_O - 28 Da]^+^ |

N.D. stands for not detected
