# Supplementary material for: Isoprenoid Alcohols are Susceptible to Oxidation with Singlet Oxygen and Hydroxyl Radicals
Source: Lipids. 2015 Dec 30;51:229–44. doi: 10.1007/s11745-015-4104-y (PMC4735226; doi:10.1007/s11745-015-4104-y)
Supplement: Supplementary file 6 — Supplementary material 6 (DOCX 71 kb) [file 11745_2015_4104_MOESM6_ESM.docx]

Supplemental Table 2. Pren-10 and its oxidized standards (Prenal-10 and epoxides of Pren-10) - mass spectrometry analysis (ESI-MS and ESI-MS/MS). Ammoniated [M + NH_4_] or lithiated [M + Li] adducts were subjected to fragmentation analysis. MW 698.6 for Pren-10.

| Products of Pren-10 | Molecular ion  *m/z* | | |  | MS/MS analysis – daughter ions | |
| --- | --- | --- | --- | --- | --- | --- |
|  | [M + Na]^+^ | [M + Li]^+^ | [M + NH_4_]^+^ |  | *m/z* | fragmentation path |
| Pren-10  (M_P-10_) | 721.6 |  |  |  | N.D. | N.D. |
|  |  | 705.6 |  |  | 687.5  619.5  551.5  483.5  415.1  347.1  279.1  211.1  143.1 | [M_P-10_ + Li - H_2_O]^+^  [M_P-10_ + Li - H_2_O - C_5_H_8_]^+^  [M_P-10_ + Li - H_2_O - 2C_5_H_8_]^+^  [M_P-10_ + Li - H_2_O - 3C_5_H_8_]^+^  [M_P-10_ + Li - H_2_O - 4C_5_H_8_]^+^  [M_P-10_ + Li - H_2_O - 5C_5_H_8_]^+^  [M_P-10_ + Li - H_2_O - 6C_5_H_8_]^+^  [M_P-10_ + Li - H_2_O - 7C_5_H_8_]^+^  [M_P-10_ + Li - H_2_O - 8C_5_H_8_]^+^ |
| Prenal-10  (M_P-10-al_) | 719.6 |  |  |  | N.D. | N.D. |
|  |  |  | 714.8 |  | 697.9  679.8  651.8 | [M_P-10-al_ + NH_4_ - NH_3_]  [M_P-10-al_ + NH_4_ - NH_3_ - H_2_O]^+^  [M_P-10-al_ + NH_4_ - NH_3_ - H_2_O - 28 Da]^+^ |
| Pren-10 monoepoxide  (M_P-10-epo_) | 737.6 |  |  |  | N.D. | N.D. |
|  |  | 721.6 |  |  | 703.6  685.6  703.6  635.5  567.5  499.4  431.4  363.1  295.1  227.1  159.0  583.5  515.0  447.0  379.0  311.0  243.0  175.0 | path A  [M_P-10-epo_ + Li - H_2_O]^+^  [M_P-10-epo_ + Li - 2H_2_O]^+^  path B  [M_P-10-epo_ + Li - H_2_O]^+^  [M_P-10-epo_ + Li - H_2_O - C_5_H_8_]^+^  [M_P-10-epo_ + Li - H_2_O - 2C_5_H_8_]^+^  [M_P-10-epo_ + Li - H_2_O - 3C_5_H_8_]^+^  [M_P-10-epo_ + Li - H_2_O - 4C_5_H_8_]^+^  [M_P-10-epo_ + Li - H_2_O - 5C_5_H_8_]^+^  [M_P-10-epo_ + Li - H_2_O - 6C_5_H_8_]^+^  [M_P-10-epo_ + Li - H_2_O - 7C_5_H_8_]^+^  [M_P-10-epo_ + Li - H_2_O - 8C_5_H_8_]^+^  path C  [M_P-10-epo_ + Li - H_2_O - C_5_H_8_]^+^  [M_P-10-epo_ + Li - H_2_O - 2C_5_H_8_]^+^  [M_P-10-epo_ + Li - H_2_O - 3C_5_H_8_]^+^  [M_P-10-epo_ + Li - H_2_O - 4C_5_H_8_]^+^  [M_P-10-epo_ + Li - H_2_O - 5C_5_H_8_]^+^  [M_P-10-epo_ + Li - H_2_O - 6C_5_H_8_]^+^  [M_P-10-epo_ + Li - H_2_O - 7C_5_H_8_]^+^ |
|  |  |  | 732.5 |  | 714.5  697.5  679.4 | path D  [M_P-10-epo_ + NH_4_ - H_2_O]^+^  [M_P-10-epo_ + NH_4_ - H_2_O - NH_3_]^+^  [M_P-10-epo_ + NH_4_ - H_2_O - NH_3_ - H_2_O]^+^ |
| Pren-10 diepoxide  (M_P-10-diepo_) | 753.5 |  |  |  | N.D. | N.D. |
|  |  | 737.5 |  |  | 719.5  701.5  683.5  615.4  719.5  651.4  583.4  515.4  447.3  379.2  311.0  243.1  175.0  635.5  567.5  499.4  431.4  363.1  295.1  227.1  159.0  707.6  689.6 | path A  [M_P-10-diepo_ + Li - H_2_O]^+^  [M_P-10-diepo_ + Li - 2H_2_O]^+^  [M_P-10-diepo_ + Li - 3H_2_O]^+^  [M_P-10-diepo_ + Li - 2H_2_O - C_5_H_8_]^+^  path B  [M_P-10-diepo_ + Li - 2H_2_O - C_5_H_8_]^+^  [M_P-10-diepo_ + Li - 2H_2_O - 2C_5_H_8_]^+^  [M_P-10-diepo_ + Li - 2H_2_O - 3C_5_H_8_]^+^  [M_P-10-diepo_ + Li - 2H_2_O - 4C_5_H_8_]^+^  [M_P-10-diepo_ + Li - 2H_2_O - 5C_5_H_8_]^+^  [M_P-10-diepo_ + Li - 2H_2_O - 6C_5_H_8_]^+^  [M_P-10-diepo_ + Li - 2H_2_O - 7C_5_H_8_]^+^  [M_P-10-diepo_ + Li - 2H_2_O - 8C_5_H_8_]^+^  [M_P-10-diepo_ + Li - 2H_2_O - 9C_5_H_8_]^+^  path C  [M_P-10-diepo_ + Li - 2H_2_O - C_5_H_8_]^+^  [M_P-10-diepo_ + Li - 2H_2_O - 2C_5_H_8_]^+^  [M_P-10-diepo_ + Li - 2H_2_O - 3C_5_H_8_]^+^  [M_P-10-diepo_ + Li - 2H_2_O - 4C_5_H_8_]^+^  [M_P-10-diepo_ + Li - 2H_2_O - 5C_5_H_8_]^+^  [M_P-10-diepo_ + Li - 2H_2_O - 6C_5_H_8_]^+^  [M_P-10-diepo_ + Li - 2H_2_O - 7C_5_H_8_]^+^  [M_P-10-diepo_ + Li - 2H_2_O - 8C_5_H_8_]^+^  path D  [M_P-10-diepo_ + Li - H_2_O]^+^  [M_P-10-diepo_ + Li - 2H_2_O]^+^ |
| Pren-10 triepoxide  (M_P-10-triepo_) | 769.6 |  |  |  | N.D. | N.D. |
|  |  | 753.6 |  |  | 735.5  717.5  699.6  735.5  667.4  599.4  531.4  463.3  395.2  327.1  259.1  191.0  651.4  583.4  515.4  447.3  379.2  311.0  243.0  175.0 | path A  [M_P-10-triepo_ + Li - H_2_O]^+^  [M_P-10-triepo_ + Li - 2H_2_O]^+^  [M_P-10-triepo_ + Li - 3H_2_O]^+^  path B  [M_P-10-triepo_ + Li - H_2_O - C_5_H_8_]^+^  [M_P-10-triepo_ + Li - H_2_O - 2C_5_H_8_]^+^  [M_P-10-triepo_ + Li - H_2_O - 3C_5_H_8_]^+^  [M_P-10-triepo_ + Li - H_2_O - 4C_5_H_8_]^+^  [M_P-10-triepo_ + Li - H_2_O - 5C_5_H_8_]^+^  [M_P-10-triepo_ + Li - H_2_O - 6C_5_H_8_]^+^  [M_P-10-triepo_ + Li - H_2_O - 7C_5_H_8_]^+^  [M_P-10-triepo_ + Li - H_2_O - 8C_5_H_8_]^+^  [M_P-10-triepo_ + Li - H_2_O - 9C_5_H_8_]^+^  path C  [M_P-10-triepo_ + Li - H_2_O - C_5_H_8_]^+^  [M_P-10-triepo_ + Li - H_2_O - 2C_5_H_8_]^+^  [M_P-10-triepo_ + Li - H_2_O - 3C_5_H_8_]^+^  [M_P-10-triepo_ + Li - H_2_O - 4C_5_H_8_]^+^  [M_P-10-triepo_ + Li - H_2_O - 5C_5_H_8_]^+^  [M_P-10-triepo_ + Li - H_2_O - 6C_5_H_8_]^+^  [M_P-10-triepo_ + Li - H_2_O - 7C_5_H_8_]^+^  [M_P-10-triepo_ + Li - H_2_O - 8C_5_H_8_]^+^ |

N.D. stands for not detected

**Pren-10 and its oxidized standards (Prenal-10 and epoxides of Pren-10) - mass spectrometry analysis (ESI-MS and ESI-MS/MS) - comments to the results shown in Supplemental Table 2.**

# The CID spectrum of lithiated P-10 monoepoxide (*m/z* 721.6) revealed the fragmentation path with the elimination of two water molecules (*m/z* 703.6, 685.6) and, as it happened in the CID spectrum with lithium salt addition, subsequent isoprene residues (in the *m/z* range: 159.0 - 635.5 and 583.5 - 175.0) (Supplemental Table 2). Additionally, ammoniated P-10 monoepoxide parent ion *m/z* 732.5 showed the loss of two water molecules (*m/z* 715.4 and 679.4). The fragmentation path of P-10 monoepoxide is analogous to that of monoepoxide of the Prenol-2 (Supplemental Table 1).

# The fragmentation spectrum of P-10 diepoxide and triepoxide was performed with lithium salt addition. In the spectrum of P-10 diepoxide (*m/z* 737.5) the loss of three subsequent water molecules was observed (*m/z* 719.5, 701.5, 683.5), in accordance with the presence of two additional oxygen atoms in the molecule of P-10. The fragment ions corresponding to the loss of the subsequent isoprene residues were also found (in the range of *m/z* 159.0 - 635.5 and 175.0 - 719.0).

In the fragmentation spectrum of P-10 triepoxide (*m/z* 753.6) the elimination of three water molecules was noticed (*m/z* 735.5, 717.5, 699.6). Moreover, signals confirming the polyprenyl chain structure by the loss of isoprene residues with 68 Da were also found (*m/z* range: 735.5 - 191.0 and 651.4 - 175.0).
