# Supplementary material for: Isoprenoid Alcohols are Susceptible to Oxidation with Singlet Oxygen and Hydroxyl Radicals
Source: Lipids. 2015 Dec 30;51:229–44. doi: 10.1007/s11745-015-4104-y (PMC4735226; doi:10.1007/s11745-015-4104-y)
Supplement: Supplementary file 7 — Supplementary material 7 (DOCX 60 kb) [file 11745_2015_4104_MOESM7_ESM.docx]

Supplemental Table 3. LC/MS analysis of Pren-10 epoxides.

| Collected fractions | Time window  [min] | Molecular ion  *m/z* | | Epoxides of Pren-10 |
| --- | --- | --- | --- | --- |
|  |  | [M + Na]^+^ | [M + Li]^+^ |  |
| 1 | 17.5 - 24.8 | 769.9* | 801.9 | hexaepoxide |
|  |  |  | 785.9 | pentaepoxide |
|  |  |  | 769.9 | tetraepoxide |
|  |  |  | 753.9 | triepoxide |
| 2 | 25.8 - 26.3 | 737.8; 753.8 | 721.9; 737.9 | monoepoxide and diepoxide |
| 3 | 26.4 - 26.9 | 737.8; 753.8 | 721.9; 737.9 | monoepoxide and diepoxide |
| 4 | 27.7 - 28.1 | 737.8; 753.8 | 721.9; 737.9 | monoepoxide and diepoxide |
| 5 | 28.3 - 28.7 | 737.8; 753.8 | 721.9; 737.9 | monoepoxide and diepoxide |
| 6 | 28.8 - 29.3 | 737.8; 753.8 | 721.9; 737.9 | monoepoxide and diepoxide |
| 7 | 29.5 - 29.9 | 737.8 | 721.9 | monoepoxide |
| 8 | 30.3 - 30.7 | 737.8 | 721.9 | monoepoxide |
| 9 | 31.4 - 31.8 | 737.8 | 721.9 | monoepoxide |
| 10 | 34.1 - 34.5 | 737.8 | 721.9 | monoepoxide |

* low intensity signals corresponding to oligoepoxides
