# Supplementary material for: Isoprenoid Alcohols are Susceptible to Oxidation with Singlet Oxygen and Hydroxyl Radicals
Source: Lipids. 2015 Dec 30;51:229–44. doi: 10.1007/s11745-015-4104-y (PMC4735226; doi:10.1007/s11745-015-4104-y)
Supplement: Supplementary file 8 — Supplementary material 8 (DOCX 62 kb) [file 11745_2015_4104_MOESM8_ESM.docx]

Supplemental Table 4. Products of oxidation of Pren-10 (MW 698.6) after hydrogen peroxide treatment at 55°C after 1 h - mass spectrometry analysis (ESI-MS and ESI-MS/MS). Ammoniated adducts [M + NH_4_] were subjected to fragmentation analysis.

| Products of Pren-10 | Molecular ion  *m/z* | |  | MS/MS analysis – daughter ions | | Number of additional oxygen atoms  enriched in Prenol-10 molecule  [M_Prenol-10_ + n *x* O + Na]^+^  n |
| --- | --- | --- | --- | --- | --- | --- |
|  | [M + Na]^+^ | [M + NH_4_]^+^ |  | *m/z* | fragmentation path |  |
| Product_P-10_ No 1 | 737.8 | 732.5 |  | 697.5  679.5 | [M + NH_4_ - NH_3_ - H_2_O]^+^  [M + NH_4_ - NH_3_ - 2H_2_O]^+^ | 1 |
| Product_P-10_ No 2 | 753.9 | 748.6 |  | 730.6,  695.6  679.6 | [M + NH_4_ - NH_3_ - H_2_O]^+^  [M + NH_4_ - NH_3_ - 2H_2_O]^+^  [M + NH_4_ - NH_3_ - 2H_2_O - 16 Da]^+^ | 2 |
| Product_P-10_ No 3 | 769.8 | 764.9 |  | n.p. | n.p. | 3 |
| Product_P-10_ No 4 | 785.8 | 780.9 |  | n.p. | n.p. | 4 |
| Product_P-10_ No 5 | 801.9 | 796.9 |  | n.p. | n.p. | 5 |
| Product_P-10_ No 6 | 817.8 | 812.9 |  | n.p. | n.p. | 6 |
| Product_P-10_ No 7 | 833.9 | 828.9 |  | n.p. | n.p. | 7 |
| Product_P-10_ No 8 | 849.8 | 844.8 |  | n.p. | n.p. | 8 |
| Product_P-10_ No 9 | 865.9 | 860.8 |  | n.p. | n.p. | 9 |
| Product_P-10_ No 10 | 881.9 | 876.8 |  | n.p. | n.p. | 10 |

n.p. – not performed

**Products of oxidation of Pren-10 after hydrogen peroxide treatment at 55°C after 1 h – comments to Supplemental Table 6.**

ESI-MS analysis of the reaction mixture showed signals corresponding to sodiated ions of P-10 containing additional oxygen atoms (Product_P-10_ No 1 – 10; Δ *m/z* from 1 x 16 Da to 10 x 16 Da,) already after 1h (Supplemental Figure 1).

The CID spectrum of the ammoniated ions of Product_P-10_ No 1 and 2 (*m/z* 732.5, 748.6) is summarized in Supplemental Table 6. The ion at *m/z* 732.5 showed loss of two water molecules (*m/z* 697.5, 679.5) while *m/z* 748.6 also showed loss of two water molecules (*m/z* 730.6, 695.6) and a 16 Da fragment (*m/z* 679.6). This observation indicated the presence of two and three additional oxygen atoms in the structure of Product_P-10_ No 1 and 2, respectively. These fragmentation patterns were similar but not analogous to those observed for the model P-10 epoxide derivatives. Moreover, the collision energy (CE) required for fragmentation of Product_P-10_ No 1 and 2 was set at 45 eV while for model P-10 epoxides CE 30 eV was sufficient.
