# Supplementary material for: Isoprenoid Alcohols are Susceptible to Oxidation with Singlet Oxygen and Hydroxyl Radicals
Source: Lipids. 2015 Dec 30;51:229–44. doi: 10.1007/s11745-015-4104-y (PMC4735226; doi:10.1007/s11745-015-4104-y)
Supplement: Supplementary file 9 — Supplementary material 9 (DOCX 62 kb) [file 11745_2015_4104_MOESM9_ESM.docx]

Supplemental Table 5. Products of oxidation of Pren-10 (MW 698.6) and Pren-2 (MW 154.2) with hydroxyl radicals generated under ultrasounds - mass spectrometry analysis (ESI-MS).

| Products of  Pren-10 | Products of  Pren-2 | Molecular ion  *m/z* | |  | Number of additional oxygen atoms  enriched in isoprenoid molecule  n | |
| --- | --- | --- | --- | --- | --- | --- |
|  |  | [M_P-10_ + Na]^+^ | [M_P-2_ + Na]^+^ |  | [M_Prenol-10_ + n *x* O + Na]^+^ | [M_Prenol-2_ + n *x* O + Na]^+^ |
| Product_P-10_ No 11 | Product_P-2_ No 4 | 737.8 | 193.5 |  | 1 | 1 |
| Product_P-10_ No 12 | Product_P-2_ No 5 | 753.8 | 209.5 |  | 2 | 2 |
| Product_P-10_ No 13 | Product_P-2_ No 6 | 769.8 | 225.4 |  | 3 | 3 |
| Product_P-10_ No 14 | Product_P-2_ No 7 | 785.8 | 241.4 |  | 4 | 4 |
| Product_P-10_ No 15 | - | 801.8 | - |  | 5 | - |
| Product_P-10_ No 16 | - | 817.8 | - |  | 6 | - |
| Product_P-10_ No 17 | - | 833.8 | - |  | 7 | - |
| Product_P-10_ No 18 | - | 849.8 | - |  | 8 | - |
| Product_P-10_ No 19 | - | 865.8 | - |  | 9 | - |
| Product_P-10_ No 20 | - | 881.8 | - |  | 10 | - |

**Products of oxidation of Pren-10 (MW 698.6) and Pren-2 (MW 154.2) with hydroxyl radicals generated under ultrasounds (ESI-MS) – comments to Supplemental Table 5**

Sonications of Prenol-2 and Prenol-10 in the presence of hydrogen peroxide indicated their susceptibility to oxidative degradation. ESI-MS analysis showed signals of sodiated ions of oxidized products (*m/z* corresponding to the sequential increase of the ion mass Δ *m/z* 16) both for P-2 and P-10. Four signals (*m/z* 193.5 to *m/z* 241.4 for Product_P-2_ No 4 to 7) for Prenol-2 and ten products (Product_P-10_ No 11 to 20 at the range *m/z* 737.8 ÷ *m/z* 881.8) for P-10, were detected.
